# Metformin Promotes Cholangiocyte Regeneration in Cholestatic Fibrosis via HK1‐Glycolysis‐Dependent Epigenetic Activation of LGR5

**DOI:** 10.1002/mco2.71143

**Published:** 2026-11-13

**Authors:** Yingyu Liang, Yongqin Ye, Haoran Lian, Zhejun Song, Xiaowen Bao, Yue Peng, Wenxia Zhang, Qingyu Lu, Weilu Wang, Yiling Chen, Vincent Chi Hang Lui, Fotios Sampaziotis, Paul Kwong Hang Tam, Yan Chen

**Affiliations:** ^1^ School of Pharmacy Faculty of Medicine Macau University of Science and Technology Macau China; ^2^ Faculty of Medicine Macau University of Science and Technology Macau China; ^3^ Department of Surgery Li Ka Shing Faculty of Medicine The University of Hong Kong Hong Kong China; ^4^ Wellcome–MRC Cambridge Stem Cell Institute Cambridge UK; ^5^ Precision Regenerative Medicine Research Centre Medical Sciences Division Macau University of Science and Technology Macau China

**Keywords:** acetyl‐H4K16, cholangiocyte regeneration, cholestatic liver fibrosis, HK1, metformin

## Abstract

Cholestatic liver fibrosis, characterized by impaired cholangiocyte regeneration and elevated epithelial–mesenchymal transition (EMT)‐like remodeling, lacks effective therapeutic treatment options. Although drug repurposing was explored, metformin was identified as a potential therapeutic candidate for cholestasis, as it is a safe and cost‐effective agent with established hepatic bioavailability. Using bile duct ligation and *Mdr2* ^−/−^ mouse models, we demonstrated metformin promoted cholangiocyte regeneration and attenuated EMT‐like remodeling. Transcriptomic analysis identified HK1‐mediated glycolysis as a key downstream pathway upregulated by metformin. Cholangiocyte‐specific *Hk1* conditional knockout mice demonstrated that HK1 is essential for the antifibrotic and regenerative effects of metformin on cholangiocytes. Mechanistically, metformin enhanced HK1‐mediated glycolysis in cholangiocyte organoids, increasing acetyl‐CoA and H4K16ac enrichment at the *Lgr5* promoter, thereby promoting *Lgr5* transcription and expression. Inhibition of ACLY activity or H4K16ac deposition abolished metformin‐induced LGR5 upregulation and organoid regeneration, indicating H4K16ac is functionally required for *Lgr5* transcription and the associated regenerative response. These findings were corroborated in organoids derived from biliary atresia patients. Collectively, our findings demonstrated metformin activates the HK1–glycolysis–acetyl‐CoA–H4K16ac–LGR5 axis to promote cholangiocyte regeneration. Given its established safety profile and cost‐effectiveness, metformin represents a promising candidate for further preclinical and translational investigation as a therapeutic strategy for cholestasis.

## Graphical abstract

Metformin activates the HK1–glycolysis–acCoA–H4K16ac–LGR5 axis to promote cholangiocyte regeneration and suppress EMT‐like phenotypes in cholestatic liver fibrosis. Mechanistically, metformin upregulates HK1‐driven glycolysis, increasing acetyl‐CoA availability for H4K16ac enrichment at the *Lgr5* promoter, which transcriptionally activates LGR5. This epigenetic reprogramming restores regenerative capacity, alleviating fibrotic injury.

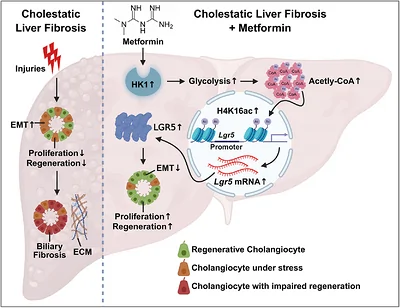

## 1 Introduction

Cholangiopathies comprise a spectrum of hepatobiliary disorders characterized by an impaired proliferative capacity in cholangiocytes. This results in progressive ductopenia due to an imbalance between apoptotic activity and regenerative proliferation, accompanied by progressive biliary fibrosis [1]. Primary biliary cholangitis (PBC), primary sclerosing cholangitis (PSC), and biliary atresia (BA) are common progressive cholangiopathies that can lead to cirrhosis, liver failure, and, in some cases, hepatocellular carcinoma in the absence of effective therapeutic intervention [2, 3, 4]. Although considerable progress has been made in understanding cholestatic liver diseases, effective therapeutic options remain limited. This unmet clinical need underscores the urgency of developing therapies that enhance cholangiocyte regeneration and slow the progression of biliary fibrosis. However, the development of novel therapeutics for cholestatic liver fibrosis is often constrained by high costs, lengthy timelines, and the substantial scientific, regulatory, and industrial challenges associated with translating new drugs from bench to bedside. Drug repurposing offers a promising strategy for the rapid advancement of cholestatic liver fibrosis treatment by leveraging safe, well‐characterized, and relatively affordable agents.

Metformin (1,1‐dimethylbiguanide hydrochloride) is the most widely prescribed antihyperglycemic agent and remains a first‐line pharmacological therapy for newly diagnosed type 2 diabetes mellitus (T2DM) [5, 6]. Previous studies have identified the liver as the major site of metformin action, where it regulates hepatic glucose production [7, 8] and modulating both the innate and adaptive immune systems [9] through both AMP‐activated protein kinase (AMPK)‐dependent and AMPK‐independent pathways. Metformin also exhibits hepatoprotective effects in animal models of metabolic dysfunction‐associated steatosis liver disease (MASLD), reducing hepatic steatosis, inflammatory responses, fibrotic progression [10, 11], as well as intrahepatic fat accumulation and serum transaminase levels [12, 13]. Building on its established efficacy and safety profile in T2DM, metformin is increasingly being investigated for repurposing across a broad range of pathological conditions, including cancer [14], age‐related diseases [15], and inflammatory and immune‐mediated diseases [16]. Although the hepatoprotective effects of metformin have been extensively investigated, it precise mechanisms of action and therapeutic potential in fibrotic cholangiopathies remain poorly understood.

Furthermore, in the absence of injury, the hepatic epithelium is maintained through slow, compartment‐specific turnover of ductal epithelial cells [17]. Following injury, regeneration and fibrosis are initiated by a shared cascade of injury‐induced cellular and molecular events, including inflammation [18], that diverge according to the duration and persistence of the underlying tissue damage [19]. Recent studies have demonstrated that leucine‐rich repeat‐containing G‐protein coupled receptor 5 (LGR5), a key component of the Wnt signaling pathway, serves as a robust biomarker of actively proliferating stem cells in the intestinal epithelium [20], gastric mucosa [21], hair follicles [22], mammary glands [23], and liver [24]. LGR5^+^ liver stem cells constitute a minor cellular population that is thought to reside near the portal triad in a quiescent state and become activated in response to hepatic injury [13, 25]. Following carbon tetrachloride (CCl4)‐induced injury, Lgr5‐LacZ^+^ cells emerge in the peribiliary region, concomitant with robust activation of Wnt signaling [24]. Additionally, lineage‐tracing studies using a novel *Lgr5‐IRES‐CreERT2* knock‐in allele have demonstrated that damage‐induced Lgr5^+^ cells can contribute to bile duct formation in vivo [24]. These findings suggest that regeneration mediated by LGR5^+^ cells represents an important mechanism for maintaining bile duct integrity and promoting regeneration under pathological conditions.

In this study, we evaluated the therapeutic effects of repurposed metformin on liver fibrosis and cholangiocyte regeneration in bile duct ligation (BDL) and *Mdr2* ^−/−^ mouse models, as well as patient‐derived organoids. Transcriptomic analysis and immunostaining identified the HK1–glycolysis pathway as a key downstream mediator of metformin's effects. The functional relevance of HK1 was further demonstrated in clinical samples from biliary atresia patients and in Krt19‐CreERT2*Hk1* ^fl/fl^ mice, in which HK1 deficiency abrogated the protective effects of metformin. In the present study, CUT&Tag and ChIP‐qPCR analyses mechanistically revealed that metformin promotes HK1–glycolysis‐dependent enrichment of H4K16ac at the *Lgr5* promoter, thereby increasing LGR5 expression and promoting cholangiocyte regeneration. Inhibition of ACLY or H4K16ac deposition abolished metformin‐induced LGR5 upregulation and organoid regeneration in a concentration‐dependent manner, supporting H4K16ac is functionally required for the regenerative response. Collectively, these findings provide new mechanistic insights into the therapeutic actions of metformin in cholestatic liver fibrosis and identify the HK1–glycolysis–H4K16ac–LGR5 axis as a potential target for therapeutic intervention.

## 2 Results

### 2.1 Metformin Improved Cholestatic Liver Injury and Fibrosis in Cholestatic Mice

To evaluate the effect of metformin (Met) on cholestatic liver diseases (CLD) development, we established a CLD‐like mouse model using common BDL and *Mdr2* ^−/−^ mice and administered metformin concurrently via intraperitoneal injection (Figure 1A). Met significantly improved the survival rate of BDL mice (Figure 1B, *p* < 0.05), and alleviated the cholestatic jaundice (Figure 1C), and reduced the elevated liver‐to‐body weight ratio (LW/BW) associated with cholestasis (Figure 1D, *p* < 0.05). Additionally, serological experiments showed Met improved liver function in both models (Figure 1E, F, all *p* < 0.05). Notably, Met attenuated excessive peri‐portal extracellular matrix (ECM) deposition, as indicated by Sirius red and Masson's trichrome staining (Figure 1G, H, *p* < 0.001), and IF staining for fibronectin 1 (FN1), vimentin, collagen type I α 1 (COL1A1), and α smooth muscle actin (αSMA) (Figure 1I, J, all *p* < 0.001). Furthermore, proliferative marker KI67 was also increased in the cholangiocytes of metformin‐treated mice (Figure 1K–L, *p* < 0.05). Overall, these findings demonstrated metformin efficiently protected against cholestatic liver injury and fibrosis.

**FIGURE 1:**
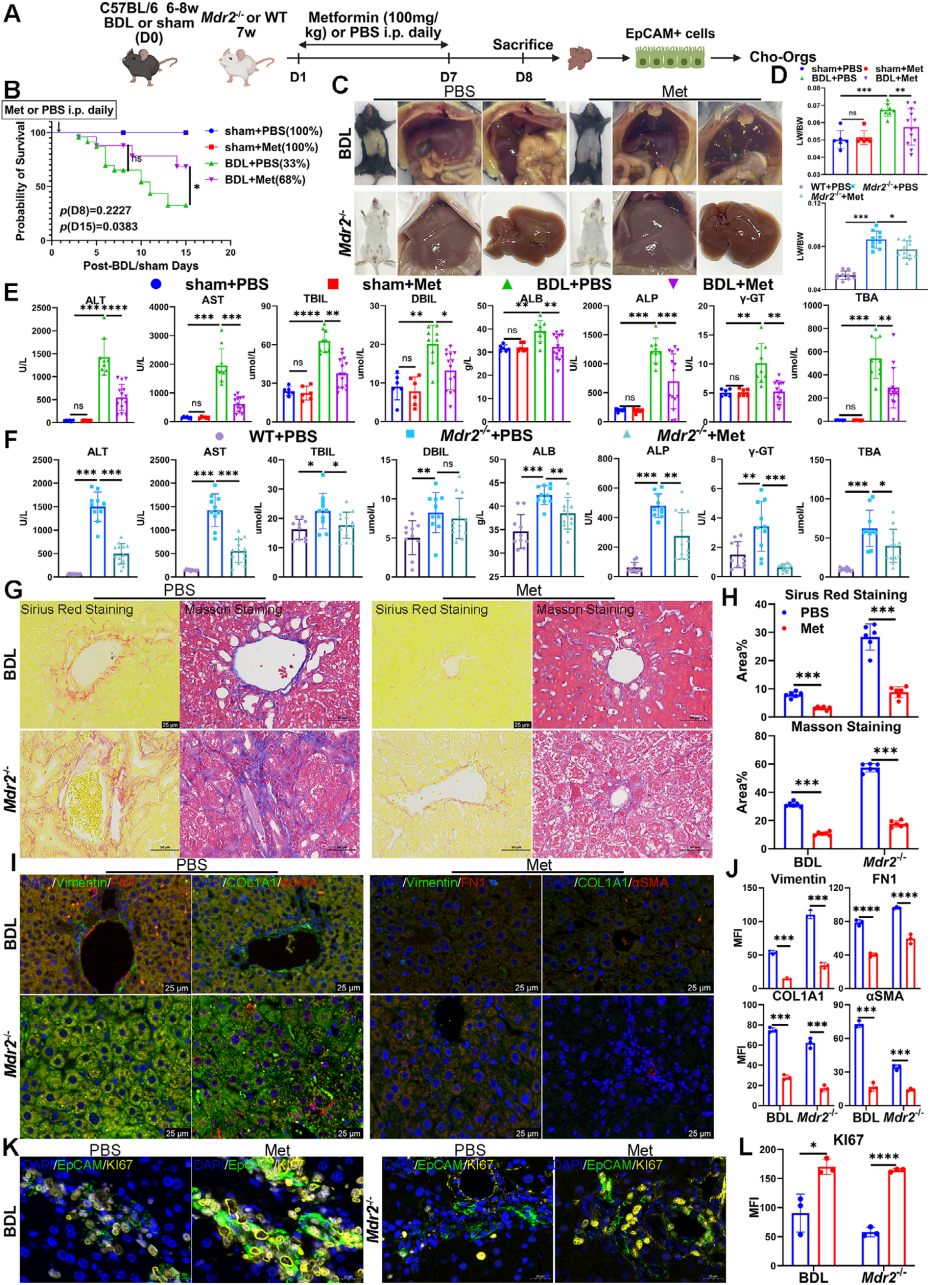
**Metformin improves cholestatic liver injury and fibrosis in cholestatic mice**. (A) Schematic of metformin treatment on BDL and *Mdr2* ^−/−^ mice. (B) Survival analysis of metformin‐treated BDL mice. (C) Representative images of metformin‐treated BDL and *Mdr2* ^−/−^ mice. (D) The ratio of liver weight and body weight. (E and F) The serum levels of liver function test of metformin‐treated BDL and *Mdr2* ^−/−^ mice. (G and H) Sirius Red, Masson staining, and quantification of liver tissue. Scale bar = 25, 50 µm. (I and J) The protein levels of and quantification of vimentin, FN1, α‐SMA, and COL1A1 in liver tissues. Scale bar = 25 µm. (K and L) The protein levels and quantification of KI67 in liver tissues. Scale bar = 200 µm. *p < 0.05, **p < 0.01, ***p < 0.001, ****p < 0.0001.

In light of the protective effects of metformin, we further characterized hepatic injury, fibrosis, and cholangiocyte proliferation in sham and BDL mice. Serum levels of alanine aminotransferase (ALT), aspartate aminotransferase (AST), alkaline phosphatase (ALP), total bile acid (TBA), total bilirubin (TBIL), direct bilirubin (DBIL), albumin (ALB), and γ‐glutamyl transferase (γ‐GT) were all progressively increased in BDL mice (Figure S1A, all *p* < 0.05). Increased ECM deposition, as evidenced by Masson's trichrome and Sirius red staining (Figure S1B,C, both *p* < 0.001), and IF staining of fibrotic markers (FN1, vimentin, COL1A1, and αSMA) (Figure S1D,E, all *p* < 0.001). In contrast, the proliferation marker KI67 decreased in EpCAM^+^ cholangiocytes in BDL mice (Figure S1F,G, *p* < 0.001).

### 2.2 Metformin Enhanced Regeneration and Glycolysis in Cholangiocyte Organoids

Next, RNA‐sequencing analysis was performed to uncover the underlying mechanisms of Met on P0 cholangiocyte organoids (Cho‐Orgs) derived from metformin‐treated BDL mice. As shown in Figure 2A, Met improved organoid growth, accompanied by elevated proliferative markers, KI67, PCNA, *Ccna2*, *Ccne1*, *Cdk1*, and *Cdk2* on Cho‐Orgs (Figure S2A–D, all *p* < 0.05). Gene set‐enrichment (GSEA) analysis further revealed significant upregulation of pathways related to mitotic recombination, DNA replication, and cell cycle following metformin treatment (Figure 2B, Figure S2E, all *p* < 0.05). In mammals, severe liver injury can induce the transdifferentiation of biliary epithelial cells into hepatocytes through an intermediate biphenotypic state characterized by the co‐expression of both biliary epithelial and hepatocyte lineage markers [26]. Met administration significantly increased cholangiocyte (CK7, CK19) and decreased hepatocyte (ALB, AFP) markers (Figure 2C,D, Figure S2F–H, all *p* < 0.05). The NOTCH signaling pathway and related genes (*Notch2*, *Jag1*, and *Hes1*), contributing to bile duct formation [27, 28] and expansion [29], were also upregulated in Met‐treated group (Figure 2E,F, all *p* < 0.05). GSEA analysis indicated Met inhibited de‐differentiated epithelial phenotype‐related pathway on Cho‐Orgs, such as TGFβ, ECM‐receptor, and downstream targets (vimentin and FN1) (Figure 2G–I, Figure S2I, all *p* < 0.05). Interestingly, Met also promoted the progenitor markers related to cholangiocyte regeneration [30, 31, 32] (LGR5, *EpCAM*, *Sox9*, *Prom1*, and *Tbx3*) in the Cho‐Orgs, among them, LGR5 showed the most significant upregulation (Figure 2J–L, Figure S2J, *p* < 0.05). These findings led us to hypothesize that LGR5 may represent as a key downstream mediator of the therapeutic effects of metformin.

**FIGURE 2:**
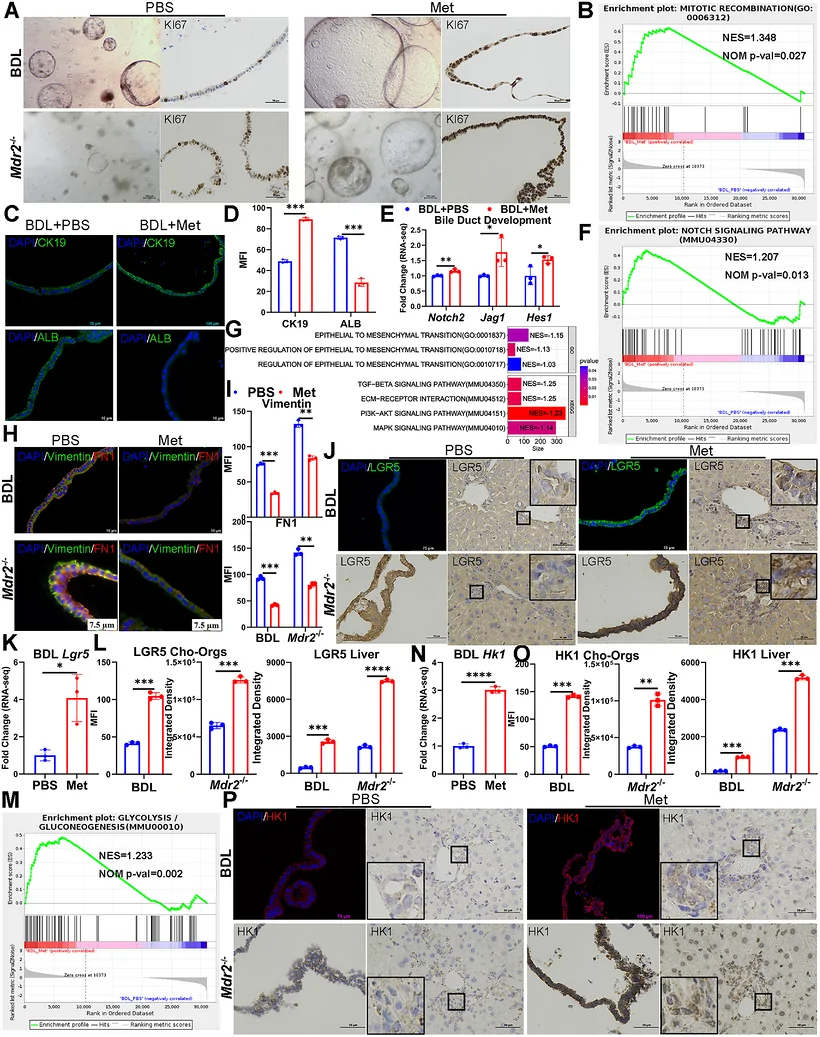
**Metformin enhances regeneration and glycolysis on Cho‐Orgs**. (A) Representative images of cell growth and KI67 staining on Cho‐Orgs derived from metformin‐treated mice. Scale bar = 50 µm. (B) GSEA analysis of MITOTIC RECOMBINATION. (C and D) The protein levels of CK19 and ALB in Cho‐Orgs section. Scale bar = 10, 100 µm. (E) The mRNA level of *Notch2*, *Jag1*, and *Hes1* in Cho‐Orgs. (F) GSEA analysis of NOTCH SIGNALING PATHWAY. (G) GSEA analysis of EMT‐like‐related pathways. (H and I) The protein levels of vimentin, FN1 in Cho‐Orgs section. Scale bar = 10 µm. (J–L) The levels of LGR5 in Cho‐Orgs and liver section. Scale bar = 50, 75 µm. (M) GSEA analysis of GLYCOLYSIS/GLUCONEOGENESIS. (N–P) The levels of HK1 in Cho‐Orgs and liver section. Scale bar = 50, 75, 100 µm. *p < 0.05, **p < 0.01, ***p < 0.001, ****p < 0.0001.

Notably, the glycolysis/gluconeogenesis pathway was also activated by metformin (Figure 2M, *p* = 0.002). The key glycolytic enzymes, HK1, *Pkm*, *Gpi1*, *Pgm1*, *Pgm2*, *Pfkp*, and *Tpi1* were significantly upregulated on Cho‐Orgs, with HK1 showing the most pronounced increase (Figure 2N–P, Figure S2L, *p* < 0.05). To explore the potential interaction between metformin and HK1, we performed in silico molecular docking analysis, which predicted a putative binding mode involving Gly593 with a binding energy of −4.32 kcal/mol (Figure S2M). These findings prompted us to investigate HK1 as a potential downstream effector of metformin's therapeutic effects.

Given the potential therapeutic effects of metformin, we further evaluated cholangiocyte regeneration, EMT‐like remodeling, and glycolytic activity in Cho‐Orgs derived from sham‐1w and BDL‐1w mice. BDL‐derived Cho‐Orgs exhibited reduced cholangiocyte marker (CK7 and CK19) expression and increased hepatocyte marker (ALB and AFP) expression, consistent with the shift toward a hepatocyte‐like phenotype (Figure S3A–C, all *p* < 0.05). An enhanced EMT‐like phenotype was also observed, as demonstrated by IF staining, which showed increased FN1 and vimentin expression (Figure S3D‐F, all *p* < 0.01). The progenitor markers, LGR5, *Sox9*, *EpCAM*, *Tbx3*, *Prom1*, and *Cd24a*, were decreased in BDL‐derived Cho‐Orgs, especially LGR5 (Figure S3G–I, all *p* <0.05), while LGR5 was increased in BDL liver (Figure S3J–K, *p* < 0.001). BDL‐derived Cho‐Orgs exhibited reduced expression of proliferation‐ and cell cycle‐associated markers (Figure S3L–N, all *p* < 0.05). Glycolytic enzyme, HK1, was also downregulated in BDL (Figure S3O–R, both *p* < 0.001). Thus, impaired regeneration, proliferation, glycolysis, and an excessive EMT‐like epithelial phenotype were observed in the BDL‐derived Cho‐Orgs.

### 2.3 Glycolysis‐Derived Acetyl‐CoA Promotes *Lgr5* Expression via H4K16ac at TSS

To further investigate the role of glycolysis in the effects of metformin, we performed GSEA analysis on bulk RNA‐seq from Cho‐Orgs derived from metformin‐treated BDL mice. GSEA revealed significant enrichment of pathways related to histone H4 acetyltransferase activity and H4‐K16 acetylation (Figure 3A,B, both *p* < 0.05). Immunohistochemistry (IHC) staining of H4K16ac further supported this finding (Figure 3C,D, *p* < 0.01), accompanied by elevated levels of the glycolytic metabolite acetyl‐CoA in serum and Cho‐Org supernatant (Figure 3E, all *p* < 0.001). Previous study showed that H4K16ac is instructive for future gene activation [33]. To investigate whether H4K16ac can serve as a core for *Lgr5* transcription, CUT&Tag sequencing analysis was conducted. A heatmap revealed increased H4K16ac enrichment at the *Lgr5* transcription starting site in Cho‐Orgs from metformin‐treated BDL mice, whereas this enrichment was not observed in the sham + metformin group (Figure 3F–G). Furthermore, H4K16ac enrichment at the *Lgr5* promoter was significantly increased in the metformin‐treated group, whereas no significant change was observed in the metformin‐treated sham group (Figure 3H). CUT&Tag qPCR further confirmed that metformin increased H4K16ac enrichment at the *Lgr5* promoter in *Mdr2* ^−/−^ mice (Figure 3I, both *p* < 0.001).

**FIGURE 3:**
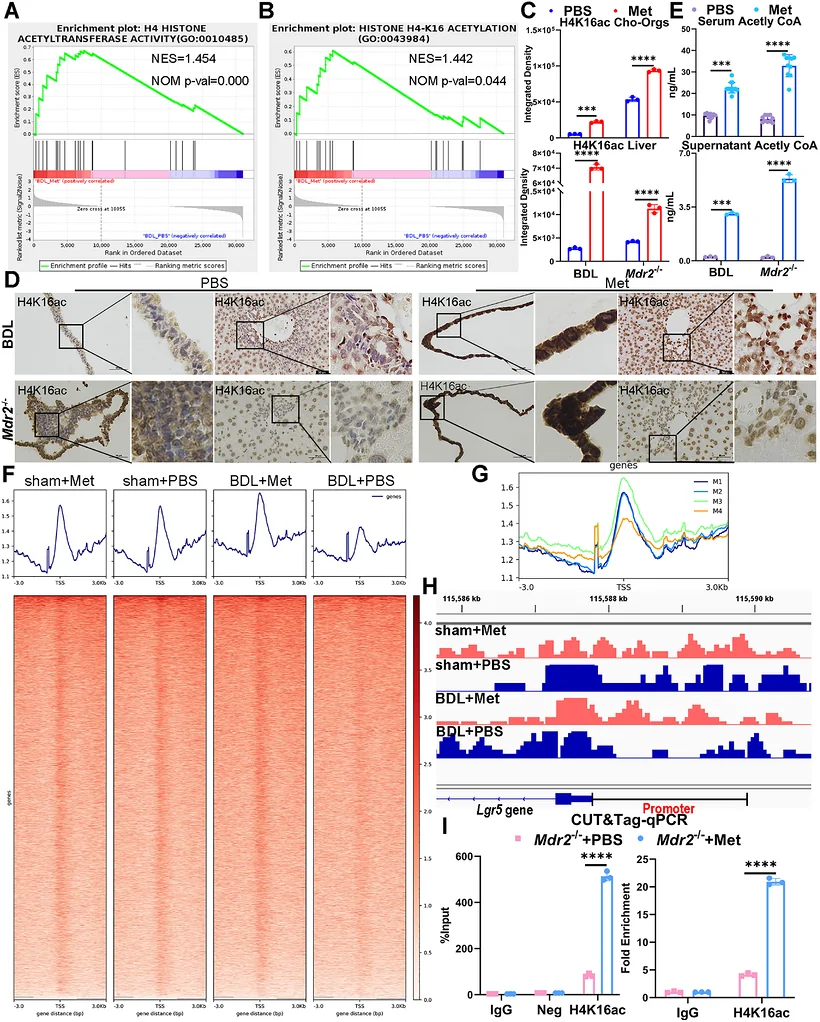
**Glycolysis‐derived acetyl‐CoA promotes *Lgr5* by enhancing H4K16ac at TSS on Cho‐Orgs**. (A and B) GSEA analysis of H4 HISTONE ACETYLTRANSFERASE ACTIVITY and HISTONE H4‐K16 ACETYLATION. (C and D) Staining and quantification of H4K16ac of Cho‐Orgs and liver section. Scale bar = 50 µm. (E) ELISA detection of acetyl‐CoA in serum and organoid supernatant. (F and G) Heatmap of the enrichment between H4K16ac and genes. M1, sham+Met; M2, sham+PBS; M3, BDL+Met; M4, BDL+PBS. (H) Visualization of H4K16ac enrichment peaks on *Lgr5* locus. (I) CUT&Tag qPCR of H4K16ac enrichment at *Lgr5* promoter. *p < 0.05, **p < 0.01, ***p < 0.001, ****p < 0.0001.

Given that metformin transcriptionally upregulated *Acly* (Figure S4A, *p* < 0.01), we next assessed whether ACLY enzymatic activity is functionally required for metformin's downstream effects. To test this, we treated metformin‐stimulated organoids (derived from BDL and *Mdr2* ^−/−^ models) with NDI‐091143. We first assessed potential non‑specific cytotoxicity by performing CCK‑8 assays following treatment with NDI‑091143 (1–20 µM) for 7 days. Although modest but statistically significant reductions in cell viability were observed at higher concentrations compared to DMSO (*p* < 0.01), viability remained above 95% across all NDI‐091143 concentrations in both organoid models, indicating minimal cytotoxicity threshold (Figure S4B). NDI‐091143 significantly reduced intracellular acetyl‐CoA levels, confirming effective inhibition of ACLY activity (Figure S4D, all *p* < 0.001). NDI‐091143 significantly inhibited metformin‐induced organoid growth, H4K16ac elevation, LGR5 upregulation, and KI67 enhancement in a concentration‐dependent manner (Figure S4C,E–J, all *p* < 0.001).

To directly test whether H4K16ac deposition is functionally required for metformin‐induced LGR5 expression and cholangiocyte regeneration, we treated metformin‐stimulated Cho‐Orgs with MG149, a MYST family histone acetyltransferase inhibitor that blocks MOF (KAT8) and Tip60 (KAT5) with modest potency. Although modest but statistically significant reductions were observed at 50 and 100 µM compared to DMSO (*p* < 0.05), viability remained above 96% across all concentrations in both models (Figure S5A–B). MG149 significantly reduced H4K16ac elevation (Figure S5D–E, all *p* < 0.001), and similarly to ACLY inhibition, inhibited metformin‐induced organoid growth, LGR5 upregulation, and KI67 enhancement in a concentration‐dependent manner (Figure S5C,F–I, all *p* < 0.001). These findings demonstrate that H4K16ac is functionally required for *Lgr5* transcription and cholangiocyte regeneration, establishing causation rather than merely correlation. These results indicated that metformin specifically promoted *Lgr5* transcription via glycolysis–acetyl‐CoA–H4K16ac axis in cholestasis.

Next, we assessed histone H4‐K16 acetylation and acetyl‐CoA level in Cho‐Orgs derived from sham and BDL group. Less H4K16ac acetylation was observed in both Cho‐Orgs and liver cholangiocytes in the BDL mice (Figure S6A,B, both *p* < 0.01). Moreover, acetyl‐CoA was reduced in BDL Cho‐Org serum and supernatant (Figure S6C, both *p* < 0.001).

### 2.4 HK1 Was Required for the Protective Effects of Metformin in Cholestatic Liver Fibrosis

To further identify the role of HK1 in cholestatic liver fibrosis and metformin response, we obtained cholangiocyte‐specific *Hk1*‐deficient mice (Krt19‐CreERT2*Hk1* ^fl/fl^, termed as *Hk1*‐CKO) by crossing *Hk1* ^fl/fl^ and Krt19‐CreERT2 mice from company, whose knockout efficiency was confirmed by IF staining (Figure S7A). Compared to littermate *Hk1* ^fl/fl^‐sham mice, *Hk1*‐CKO‐sham mice did not show significant differences in the general appearance and survival rate (Figure 4B,C, *p* > 0.05). It is also worth mentioning that although *Hk1*‐CKO‐sham mice displayed mild upregulation of TBIL, TBA in the serum, this was not enough to lead to impaired liver function (Figure S7B, *p* < 0.05).

**FIGURE 4:**
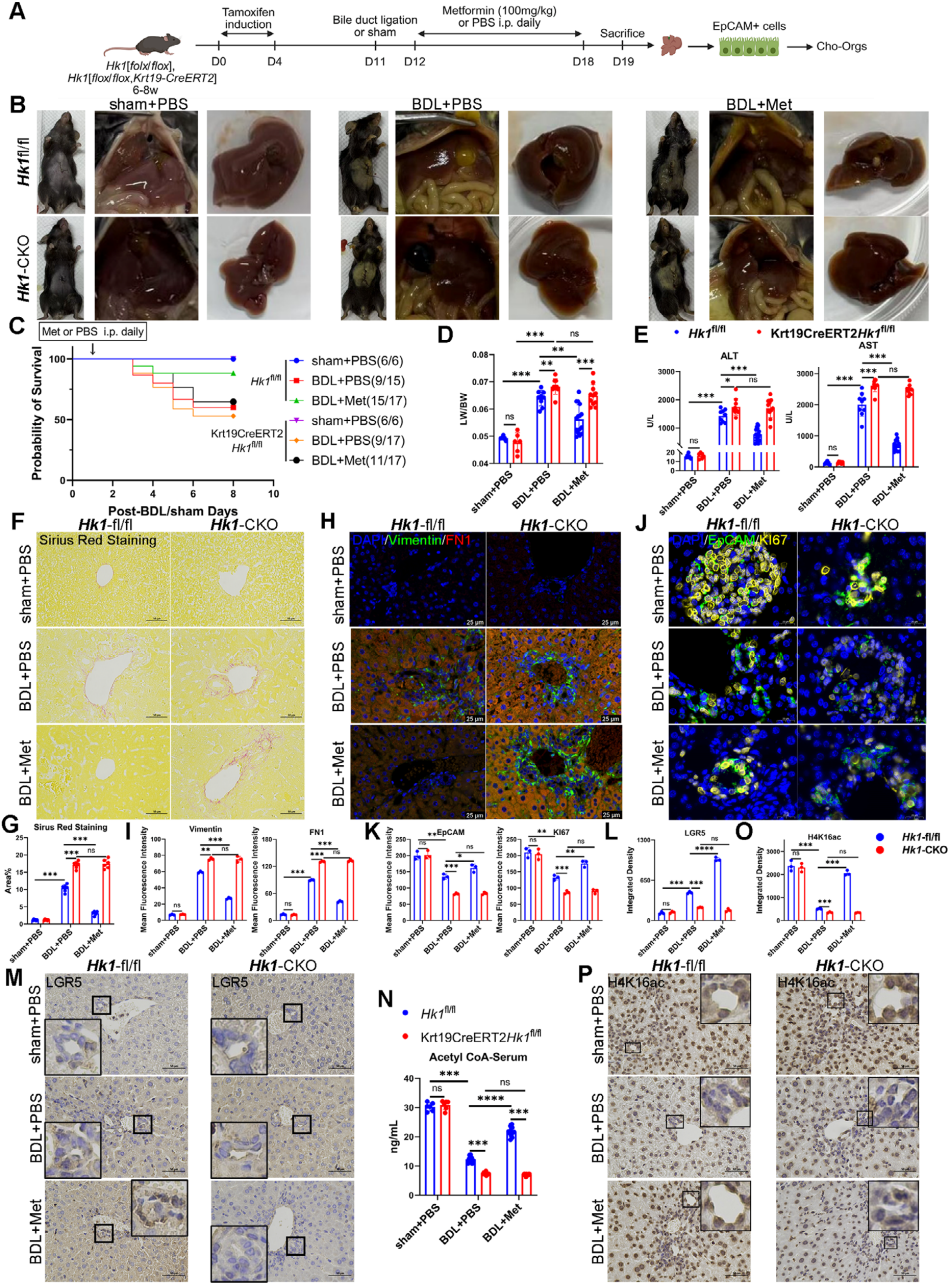
**HK1 is functionally required for the therapeutic effects of metformin in cholestatic liver fibrosis**. (A) Schematic of metformin treatment on Krt19‐CreERT2*Hk1* ^fl/fl^ mice. (B) Representative images of metformin‐treated *Hk1*‐CKO mice. (C) Survival analysis of metformin‐treated *Hk1*‐CKO mice. (D) The ratio of liver weight and body weight. (E) The serum levels of ALT and AST. (F and G) Sirius red staining and quantification of liver tissue. Scale bar = 50 µm. (H and I) The protein levels and quantification of vimentin and FN1 in liver tissues. Scale bar = 50 µm. (J and K) The protein levels and quantification of EpCAM and KI67 in liver tissues. Scale bar = 200 µm. (L and M) The protein levels and quantification of LGR5 in liver tissues. Scale bar = 50 µm. (N) The acetyl‐CoA level of serum. (O and P) The protein levels and quantification of H4K16ac in liver tissues. Scale bar = 50 µm. *p < 0.05, **p < 0.01, ***p < 0.001, ****p < 0.0001.

To determine whether HK1 is functionally required for the therapeutic effects of metformin, Krt19‐CreERT2*Hk1* ^fl/fl^ mice were administered with metformin (100 mg/kg) for 7 days following BDL (Figure 4A). Under cholestatic conditions*, Hk1‐*CKO mice exhibited more severe jaundice, an increased LW/BW, reduced survival, and exacerbated liver injury as assessed by serum biochemical analyses (Figure 4B–E, Figure S7B, all *p* < 0.05). However, the hepatoprotective effects of metformin were abolished in *Hk1*‐CKO mice, as LW/BW, survival, and serum liver function parameters remained unchanged following metformin administration (Figure 4B–E, Figure S7B, all *p* > 0.05). Severe liver dysfunction, periportal collagen deposition, and lower proliferation were observed in *Hk1*‐CKO + BDL mice compared with WT + BDL mice, as validated by hepatic histopathological examination and staining (Figure 4F–K, Figure S7C–H, all *p* < 0.01). Additionally, metformin did not improve liver fibrosis and proliferation in BDL mice when HK1 was deleted (Figure 4F–K, Figure S7C–H, *p* > 0.05). Metformin/LGR5‐assoicated regeneration in cholangiocytes was reversed by HK1 deficiency (Figure 4L–M, *p* > 0.05). Additionally, in the *Hk1*‐CKO + BDL mice, a decrease in the glycolytic product acetyl‐CoA was accompanied by a decrease in H4K16ac (Figure 4N–P, *p* < 0.05). This change was not improved by metformin administration (Figure 4N–P, *p* > 0.05). Cumulatively, these findings demonstrate that HK1 is essential for inducing the therapeutic effects of metformin in cholestatic liver fibrosis, and its deletion abolishes metformin‐induced glycolysis–acetyl‐CoA–H4K16ac–LGR5 signaling and subsequent cholangiocyte regeneration.

### 2.5 *Hk1* Deletion Disrupted Regeneration and Aggravated EMT‐Like Phenotypes in Cho‐Orgs

To further validate the effect of HK1 in Cho‐Orgs, we isolated cholangiocytes for organoid culture. *Hk1* knockout resulted in poorer cell growth, which was not ameliorated by metformin (Figure 5A). qPCR and staining further showed that elevated EMT‐like remodeling levels were observed in Cho‐Orgs in the *Hk1*‐CKO + BDL mice (Figure 5B,C, Figure S8A, *p* < 0.05). This effect was not ameliorated by metformin (Figure 5B,C, Figure S8A, *p* > 0.05). In addition to impairing cholangiocyte proliferation, as demonstrated by reduced Ki67 mRNA and protein expression (Figure 5D,E, Figure S8A, *p* < 0.01), HK1 deficiency markedly reduced LGR5 expression, and metformin failed to restore LGR5 levels following HK1 loss in Cho‐Orgs (Figure 5F,G, Figure S8A, *p* > 0.05). Furthermore, acetyl‐CoA and H4K16ac were significantly decreased in HK1‐deficienct Cho‐Orgs (Figure 5H–J, *p* < 0.05). CUT&Tag sequencing results also indicated that the H4K16ac enrichment at the *Lgr5* promoter was decreased in the *Hk1*‐CKO + BDL mice, thereby inhibiting *Lgr5* transcription (Figure 5K, Figure S8B). Therefore, HK1 is required for metformin‐mediated anti‐fibrosis and LGRB‐associated regeneration.

**FIGURE 5:**
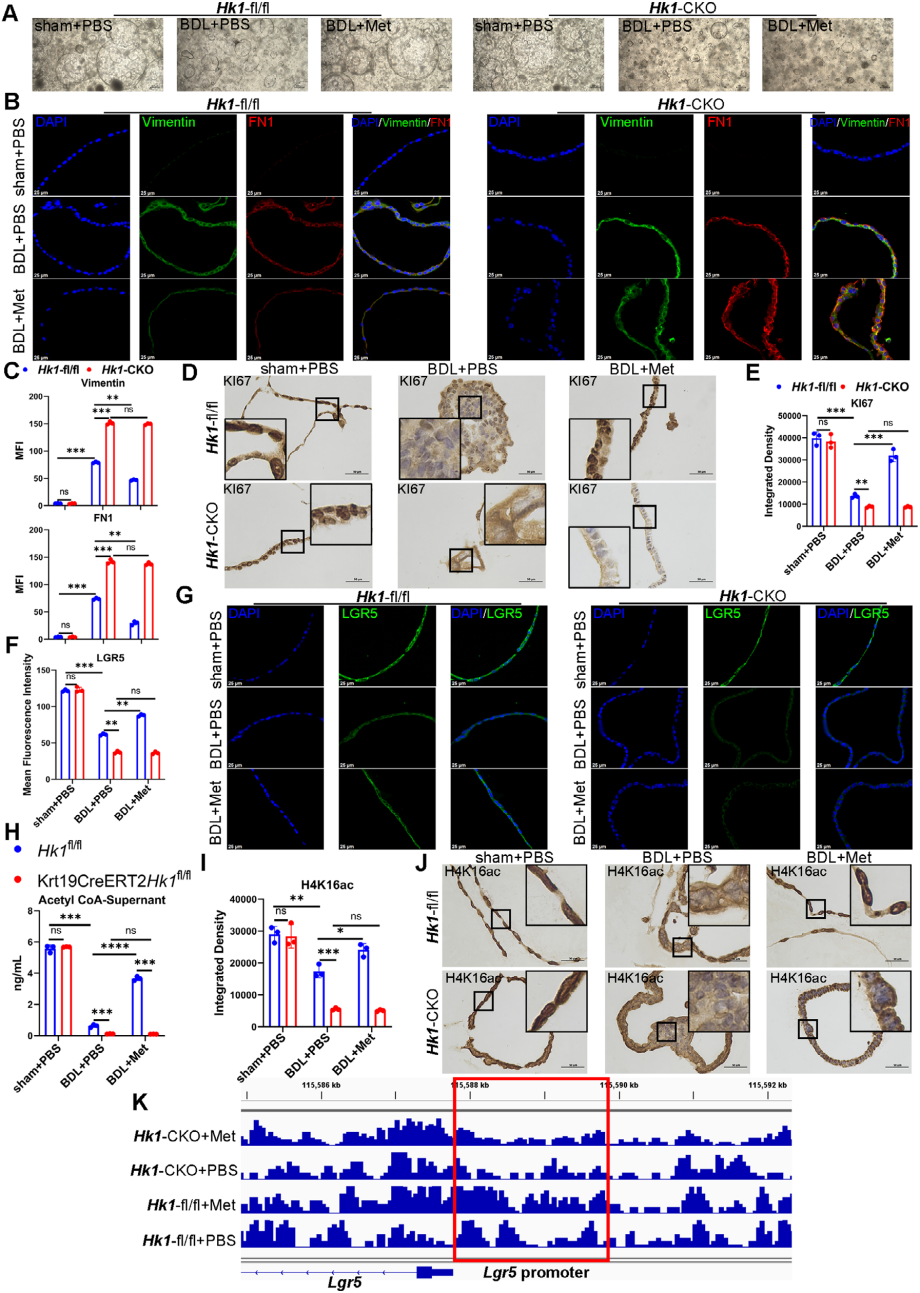
***Hk1* deletion disrupts Met‐regeneration, aggravates EMT‐like phenotypes on Cho‐Orgs**. (A) Representative images of Cho‐Orgs derived from *Hk1*‐CKO liver. Scale bar = 200 µm. (B and C) Protein levels and quantification of vimentin, FN1 of Cho‐Orgs section. Scale bar = 25 µm. (D and E) Protein levels and quantification of KI67 of Cho‐Orgs section. Scale bar = 50 µm. (F and G) Protein levels and quantification of LGR5 of Cho‐Orgs section. Scale bar = 25 µm. (H) The acetyl‐CoA level of organoid supernatant. (I and J) Protein levels and quantification of H4K16ac of Cho‐Orgs section. (K) Visualization of H4K16ac enrichment peaks on *Lgr5* locus in *Hk1*‐CKO Cho‐Orgs. Red box showed the H4K16ac enrichment peaks on *Lgr5* TSS. *p < 0.05, **p < 0.01, ***p < 0.001, ****p < 0.0001.

### 2.6 Metformin Drove BA‐Derived Cho‐Org Regeneration and Reduced EMT‐Like Remodeling via the HK1–Glycolysis–*Lgr5–*H4K16ac Axis

To investigate the therapeutic potential of metformin in human cholestatic liver diseases, we established Cho‐Orgs from three independent BA samples (*n* = 3 donors) and treated them *ex vivo* with metformin (100 µM) for 7 days. Metformin significantly promoted organoid growth in BA‐derived Cho‐Orgs (Figure 6A, Figure S9A). RNA sequencing and immunostaining further demonstrated that metformin significantly attenuated the EMT‐like phenotype (Figure 6B–D, Figures S9B and S10B, *p* < 0.01). Consistent with the finding in the mouse‐derived Cho‐Orgs, metformin enhanced expression proliferation‐associated markers, particularly KI67 (Figure 6E–G, Figures S9C and 10C, *p* < 0.05). In contrast to the Cho‐Orgs derived from the metformin‐treated BDL mice, metformin did not significantly increase cholangiocyte markers but significantly reduced hepatocyte marker expression (Figure S10A, *p* < 0.001). Additionally, metformin not only increased the LGR5 progenitor marker but also improved the glycolysis enzyme HK1 (Figure 6H–J, Figure S9D, *p* < 0.01). There was a significant increase in the glycolysis production of acetyl‐CoA in a time‐dependent manner (Figure 6K, *p* < 0.001). Meanwhile, acetyl‐H4K16 levels increased in response to metformin treatment (Figure 6L,M, Figure S9E, *p* < 0.001). To further validate the involvement of H4K16ac in *Lgr5* transcription, CUT&Tag sequencing was performed following metformin treatment. A heatmap was constructed to show that metformin treatment in BA Cho‐Orgs enhanced H4K16ac enrichment at the *Lgr5* transcription start site (Figure S10D). A significant increase in H4K16ac enrichment at the *Lgr5* promoter region was also observed (Figure 6N). These findings indicate that metformin holds potential for the treatment of human cholestatic liver diseases.

**FIGURE 6:**
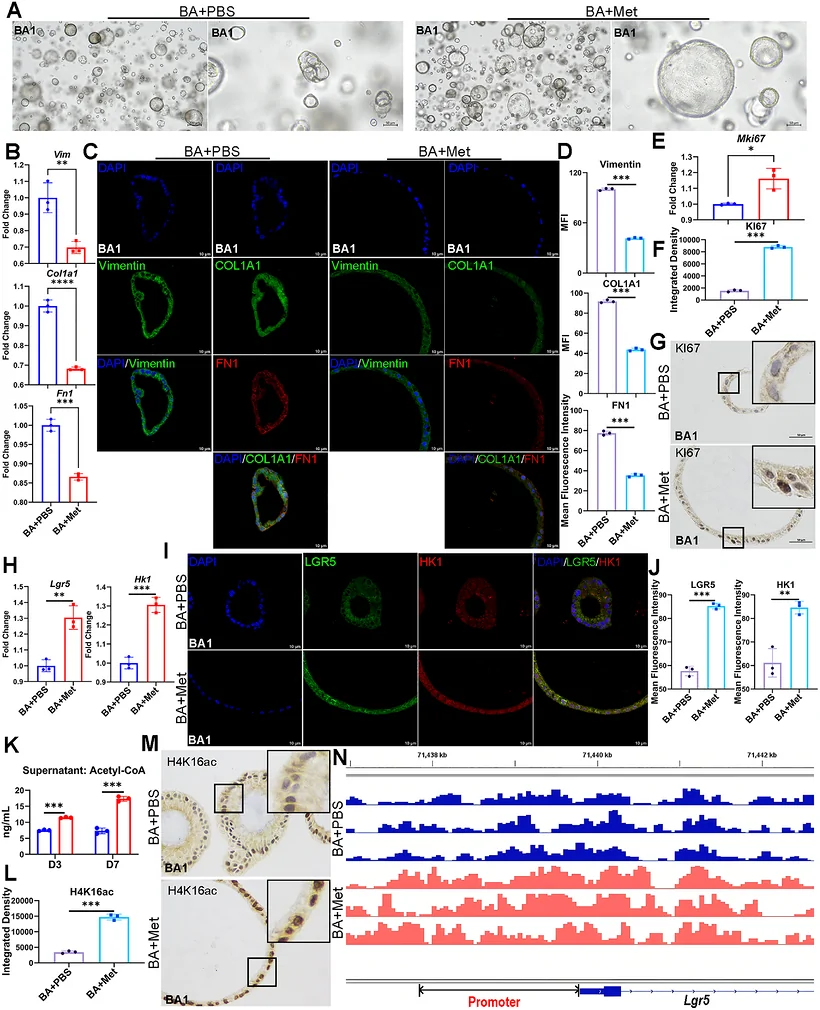
**Metformin drives BA‐derived Cho‐Orgs regeneration and reduces EMT‐like remodeling via HK1‐glycolysis–LGR5 H4K16ac axis**. (A) Representative images of metformin‐treated BA Cho‐Orgs. Scale bar = 50, 200 µm. (B–D) Expression levels and quantification of COL1A1, FN1, vimentin in metformin‐treated BA Cho‐Orgs. Scale bar = 10 µm. (E–G) Expression levels and quantification of KI67 in metformin‐treated BA Cho‐Orgs. Scale bar = 50 µm. (H–J) Expression levels and quantification of LGR5 and HK1 in metformin‐treated BA Cho‐Orgs. Scale bar = 10 µm. (K) The level of acetyl‐CoA of organoid supernatant. (L and M) Protein level and quantification of H4K16ac in metformin‐treated BA Cho‐Orgs. Scale bar = 50 µm. (N) Visualization of H4K16ac enrichment peaks on *Lgr5* locus in metformin‐treated BA Cho‐Orgs. *n* = 3 independent human BA donors (biological replicates). For each donor, technical triplicates were averaged prior to statistical analysis. All *p* values were derived from comparisons across biological replicates. *p < 0.05, **p < 0.01, ***p < 0.001, ****p < 0.0001.

## 3 Discussion

A lack of robust pre‐clinical and mechanistic evidence has thus far limited the consideration of metformin as a repurposed drug candidate for cholestatic liver fibrosis. A retrospective cohort study using the TriNetX database suggested that metformin use was associated with improved clinical outcomes in patients with PBC and DM, including lower rates of liver transplantation, all‐cause mortality, and ascites [34]. However, these findings were limited by retrospective study designs and potential confounding factors. Our study showed that metformin moderately alleviated liver damage and restored impaired cholangiocyte regeneration in both BDL and *Mdr2* ^−/−^ mice. We further demonstrated, for the first time, that HK1 deficiency exacerbated fibrotic injury in cholestatic mice and abolished the protective effects of metformin, identifying HK1 as a functionally essential mediator of metformin‐induced cholangiocyte regeneration and the consequent attenuation of liver fibrosis. Mechanistically, HK1 regulates LGR5 expression by controlling acetyl‐CoA‐dependent H4K16ac enrichment at the *Lgr5* promoter. To directly test this mechanistic model, we pharmacologically perturbed two nodes of the pathway. Cytosolic acetyl‐CoA supply was blocked using the ACLY inhibitor NDI‐091143, and H4K16ac deposition was inhibited using MG149, a MYST family HAT inhibitor that targets MOF (the primary H4K16ac‐specific acetyltransferase) and Tip60. Both interventions abolished metformin‐induced LGR5 upregulation and organoid regeneration in a concentration‐dependent manner, supporting a causal role for H4K16ac in the regenerative response. Notably, LGR5 expression was downregulated in cholestasis, whereas metformin treatment upregulated LGR5‐mediated regeneration and suppressed EMT‐related pathways, thereby attenuating cholestatic liver fibrosis. The mouse model findings were corroborated by human BA‐derived Cho‐Orgs, providing preliminary evidence for the conserved nature of this mechanism in human cells and supporting its biological plausibility.

Cholangiocytes play important functional roles in hepatic injury and contribute to the pathophysiological processes of diverse liver diseases. Although substantial evidence supports the regenerative capacity of hepatocytes, cholangiocytes can also contribute to liver regeneration when hepatocyte‐mediated repair mechanisms are compromised [35]. Accordingly, the biliary system has emerged as an important research focus of underlying hepatic regeneration and repair [36]. Research on hepatic regeneration has predominantly focused on hepatocyte biology; however, it is crucial to extend that focus to biliary system regeneration. Most forms of chronic human liver disease, particularly biliary disorders and various murine models of hepatic pathology, are characterized by significant expansion of hepatic progenitor cells (HPCs) [37]. In contrast to physiological hepatic regeneration, HPCs, including LGR5^+^, SOX9^+^, and CD44^+^ stem cells, exhibit markedly enhanced expansion and differentiation into both hepatocytes and cholangiocytes during hepatic injury and subsequent repair processes [38]. In a previous study, bipotent LGR5^+^ cells were identified in biliary ductal cells following acute CCl4‐induced hepatic injury [24]. In this study, LGR5‐expressing stem cells were not detected in the livers of sham mice, consistent with the study conducted by Huch [24], but only following liver injury induced by BDL. In this study, metformin promoted progenitor markers, including LGR5, *Sox9*, *EpCAM*, *Tbx3*, and *Prom1*; among these, LGR5 showed the most significant upregulation in BDL Cho‐Orgs. Elevated proliferation was also observed in metformin‐treated BDL Cho‐Orgs, as demonstrated by improvements in cell growth, KI67 and PCNA levels, and cell cycle markers.

*Lgr5* encodes a seven‐transmembrane G protein‐coupled receptor (GPCR) belonging to the rhodopsin subfamily [20]. LGR5 was initially identified in 2007 as a specific marker of intestinal stem cells [20]. Subsequent studies have demonstrated that *Lgr5* is also expressed in facultative stem cells that contribute to tissue regeneration following liver injury [24]. Secreted Wnt ligands and R‐spondins are critical niche signals that are typically localized in close proximity to LGR5+ stem cell compartments [39]. In the homeostatic liver, LGR5^+^ pericentral hepatocytes are likely maintained by Rspo1 or Rspo3 expression [40, 41]. NOTCH signaling regulates LGR5^+^ globose basal cells by modulating cellular proliferation and differentiation, thereby maintaining epithelial homeostasis in the normal olfactory epithelium [42]. In the present study, we identified a novel regulatory mechanism underlying LGR5 expression. CUT&Tag sequencing initially demonstrated that metformin directly enhanced *Lgr5* transcription by modulating the binding of H4K16ac at the *Lgr5* promoter in Cho‐Orgs.

Numerous stem cell types exhibit a strong dependence on anaerobic glycolysis [43]. Concurrently, glycolytic flux is substantially upregulated as evidenced by an increased expression of glucose transporters GLUT1 and GLUT3, as well as elevated enzymatic activities of HK1 and phosphofructokinase 1 (PFK1) [44]. Hexokinases (HKs) catalyze the first committed step in glucose metabolism [45, 46]. In mammals, four HK isozymes (HK1, HK2, HK3, and HK4) have been identified. Unlike HK2–4, HK1 is the predominant enzyme in glycolysis, exhibiting ubiquitous expression and widespread distribution across mammalian tissues [47]. In addition, it has been reported that HK1‐mediated glycolysis is involved in the progression of inflammation [48, 49]. Most cholangiopathies are characterized by shared pathophysiological mechanisms, encompassing inflammation, fibrogenesis, and eventually carcinogenesis [50]. However, the functions of HK1 and glycolysis in fibrotic cholangiopathy progression remain unclear. In the present study, we observed that metformin administration activated the HK1‐mediated glycolysis pathway. Our study revealed that the absence of HK1 in cholestatic mice exacerbated fibrotic liver injury and abrogated the protective effects of metformin on liver fibrosis.

Because *Hk1* was deleted specifically in *Krt19*‐lineage cholangiocytes, our genetic data primarily supported an epithelial mechanism. Fibrosis exacerbation after cholangiocyte *Hk1* deletion and the loss of metformin responsiveness in *Hk1*‐CKO mice suggest that restored cholangiocyte regenerative capacity and epithelial integrity may indirectly attenuate fibrogenic remodeling. These data do not exclude additional direct effects of metformin on hepatic stellate cells, portal fibroblasts, macrophages, or other stromal populations. However, such effects were not directly examined in the present study and will require cell type–specific analyses in future work.

Although molecular docking suggested a possible low‐to‐moderate affinity interaction between metformin and HK1, this computational prediction does not establish direct target engagement. In the absence of biochemical validation, such as CETSA, MST, ITC, or enzymatic binding assays, our findings support HK1 as a functionally required mediator of the metformin response but do not establish HK1 as a direct pharmacological target of metformin.

We also examined whether AMPK mediates metformin's effects on HK1, glycolysis, and LGR5 in cholangiocytes. Our transcriptomic data showed that the AMPK signaling pathway was not significantly altered at the transcriptional level by metformin treatment. However, we acknowledge that AMPK activation primarily occurs through post‐translational phosphorylation (Thr172), which would not be captured by a transcriptomic analysis. Thus, our findings do not formally exclude AMPK activation, and we refrain from concluding AMPK independence. Nevertheless, the absence of transcriptional changes in AMPK pathway genes may suggest that the HK1–glycolysis–H4K16ac–LGR5 axis identified in our study operates independently of AMPK transcriptional regulation. Whether AMPK phosphorylation is involved in this pathway remains an open question for future investigation.

Although our data from BDL‐derived Cho‐Orgs indicate that metformin suppresses the expression of EMT‐associated markers, we acknowledge the ongoing debate regarding the occurrence and functional significance of complete EMT in liver epithelial cells in vivo. Seminal lineage‐tracing studies have refuted the idea that cholangiocytes are directly converted into matrix‐producing fibroblasts during liver fibrosis [51]. Therefore, the metformin‐mediated effects we observed in our 3D model likely represent the suppression of a partial, reversible EMT‐like phenotypic shift associated with epithelial dysfunction and de‐differentiation rather than a definitive cell fate change. This in vitro plasticity may reflect a pathological priming state that, in the permissive organoid microenvironment, is modulated by metformin to help maintain epithelial integrity.

Glycolysis not only provides intermediates for de novo biomass synthesis but also generates metabolites that contribute to the epigenetic regulation of gene transcription [52]. In addition to being converted to lactate, pyruvate can be transported into mitochondria, where it is converted to acetyl‐CoA by the pyruvate dehydrogenase complex [53]. Notably, acetyl‐CoA is a critical metabolic substrate for protein acetylation, contributing to the post‐translational acetylation of specific amino acid residues, particularly lysine (K), on histone and non‐histone proteins [54, 55]. Wellen et al. [52] demonstrated that mammalian cells depend on ATP citrate lyase to convert mitochondrial citrate derived from glucose into nuclear acetyl‐CoA, which subsequently functions as the essential substrate for histone acetylation. Specifically, enhanced glycolytic activity leads to impaired SIRT1‐mediated deacetylation of its target histone residue H4K16 [56]. It has been reported that H4K16ac is instructive for future gene activation [33]. Our findings showed that metformin upregulated the glycolytic product acetyl‐CoA, leading to increased acetyl‐H4K16 levels at the *Lgr5* promoter and elevated LGR5 expression in Cho‐Orgs. Metformin has been reported to exert hepatoprotective effects in BDL models through GPx activation [57]. Metabolically, HK1 and GPx can be linked via the pentose phosphate pathway, which generates NADPH for glutathione reduction. However, our transcriptomic data showed that the pentose phosphate pathway was not significantly altered by metformin in Cho‐Orgs, suggesting that the GPx‐mediated antioxidant effect and our proposed HK1–glycolysis–H4K16ac epigenetic axis may represent parallel, complementary mechanisms rather than a single linear pathway. Future studies are required to determine whether HK1 exerts non‐enzymatic effects on NADPH/GSH homeostasis in cholangiocytes.

Metformin is known to inhibit mitochondrial complex I, which would constrain mitochondrial oxidative metabolism. Our transcriptomic data did not directly demonstrate complex I inhibition, nor do we claim such, as oxidative phosphorylation and the NADH/NAD+ metabolic pathways showed no significant changes. Rather, our data showed that metformin upregulated HK1 and enhanced glycolysis in cholangiocytes. We propose that this glycolytic upregulation resulted in carbon flux for cytosolic acetyl‐CoA production via the pyruvate–PDH–citrate–ACLY axis, as this is the textbook pathway connecting glucose metabolism to histone acetylation. Critically, mitochondrial and cytosolic acetyl‐CoA are located in distinct compartments with distinct functions. The acetyl‐CoA that drives H4K16ac is derived from the cytosolic pool via ACLY, not directly from mitochondria. This compartmental distinction resolves the mechanistic tension. Hence, even if complex I were inhibited, glycolysis‐derived carbon could still fuel the cytosolic acetyl‐CoA pool for epigenetic regulation. Our findings thus define a glycolysis‐to‐epigenetics axis that operates through ACLY‐mediated cytosolic acetyl‐CoA production and H4K16ac‐dependent Lgr5 transcription, independent of the mitochondrial complex I status.

We acknowledge that MG149 also inhibits Tip60, a related MYST family HAT with comparable potency. However, given that MOF is the principal H4K16ac‐specific acetyltransferase and considering the convergent evidence from the ACLY inhibition experiments upstream of the acetyl‐CoA supply, the complementary perturbations collectively support a functional requirement for H4K16ac in this pathway. We also acknowledge that acetate supplementation in *Hk1*‐CKO organoids could provide additional evidence for the metabolic‐epigenetic link by rescuing H4K16ac and LGR5 expression. However, given that acetate enters the acetyl‐CoA pool via ACSS2 rather than through the glycolysis–ACLY pathway [58, 59], such rescue would not directly restore the specific metabolic flux perturbed by HK1 deletion. Future studies using alternative carbon sources or metabolic intermediates may further delineate the substrate specificity of this epigenetic regulation.

Our findings in human BA‐derived organoids from three independent donors provide evidence that the HK1–glycolysis–H4K16ac–LGR5 axis is operative in human cholangiocytes and support the biological plausibility for the mechanisms identified in mouse models. However, several important limitations should be acknowledged. First, the small number of donors (*n* = 3) limits the generalizability of these findings and precludes a comprehensive assessment of biological variability among human samples. Second, BA is a pediatric cholestatic disease with distinct etiopathogenesis; therefore, our finding cannot be directly extrapolated to adult cholestatic diseases such as PBC or PSC. Third, the *ex vivo* organoid culture system does not fully recapitulate the complexity of the in vivo hepatic microenvironment, including interactions with immune cell and process underlying fibrotic remodeling. Future studies involving larger cohorts of human samples, including from adults with PBC and PSC, together with validation in additional preclinical animal models, will be needed to determine the translational potential of targeting the HK1–glycolysis–H4K16ac–LGR5 axis in human cholestatic liver diseases.

The metformin dose used in mice (100 mg/kg/day intraperitoneally) falls within the range commonly employed in rodent studies of hepatic injury and metabolic disease. However, this dose and route of administration do not directly recapitulate human oral therapeutic exposure. Similarly, the metformin concentration (100 µM) used in the organoids was selected to provide a biologically active exposure while avoiding millimolar concentrations in *ex vivo* culture. Although metformin can accumulate in hepatic tissue through organic cation transporters, the relationship between the concentrations used in this study and clinically achievable metformin exposure in cholangiocytes remains uncertain. Future pharmacokinetic and pharmacodynamic studies will be required to define clinically relevant dosing strategies for cholestatic liver disease.

Although protein‐level changes were supported by quantitative immunostaining and ELISA‐based measurements, western blot validation was not performed for all markers because of limited organoid material and the need to preserve spatial and cell type‐specific information. Future studies using purified cholangiocytes or expanded organoid biomass may provide additional biochemical validation.

A further limitation is that the in vivo experiments were performed in male mice only. Given known sex‐dependent differences in bile acid metabolism, inflammatory responses, and susceptibility to cholestatic injury, future studies should determine whether the metformin–HK1–LGR5 axis operates similarly in female mice.

## 4 Conclusion

In summary, we identified HK1 as a functionally essential mediator that suppresses liver fibrosis and promotes cholangiocyte regeneration, supported by preliminary evidence from human BA‐derived organoids and Krt19‐CreERT2*Hk1* ^fl/fl^ mice. We demonstrated, for the first time, that metformin repurposing ameliorates cholestatic liver fibrosis by promoting LGR5‐mediated bile duct regeneration, offering new therapeutic targets for fibrotic cholangiopathy. Mechanistically, metformin enhances glycolysis and acetyl‐CoA production, driving H4K16ac modification of the *Lgr5* promoter to facilitate its transcription. Together, these findings define a novel HK1‐dependent mechanism for the protective effects of metformin in cholestatic liver fibrosis and provide a mechanistic rationale for future studies to explore the therapeutic potential of targeting this pathway in human cholestatic diseases.

## 5 Materials and Methods

Part of the materials and methods are described in the Supporting Information.

### 5.1 Clinical Specimens

The clinical cohort was established at the Third Affiliated Hospital of Sun Yat‐sen University, and the study protocol was approved by its Research Ethics Committee (Approval Number: RG2024‐149‐01). The study retrospectively enrolled patients diagnosed with biliary atresia between January 2024 and June 2024. Due to the retrospective nature of the analysis, the requirement for individual patient consent was waived. All research was conducted in accordance with both the Declarations of Helsinki and Istanbul.

### 5.2 Animal Studies

Male C57BL/6J mice (8‐week‐old, male) were purchased from BesTest (Zhuhai, China). *Hk1* ^fl/fl^ mice on C57BL/6J background were structured by Cyagen Biosciences. Cholangiocyte‐specific *Hk1* knockout mice (*HK1*‐CKO) were generated by Cyagen Biosciences via crossing Krt19‐CreERT2 recombinase transgenic mice with *Hk1* ^fl/fl^. For the BDL experiments, 8‐week‐old male mice were randomly assigned to four experimental groups: (1) sham + PBS, (2) sham + metformin, (3) BDL + PBS, and (4) BDL + metformin. For the *Mdr2* ^−/−^ model, 8‐week‐old male *Mdr2* ^−/−^ mice and their wild‐type littermates were used. The individual mouse was considered as the experimental unit for all analyses. The mice were housed in a controlled and standard laboratory environment (temperature 20 ± 2°C, relative humidity 55 ± 10%, and a 12‐h light/12‐h dark cycle) with water and a chow diet available ad libitum. All animal experiments were performed after institutional review by the Animal Ethics Committee of the Macau University of Science and Technology (MUST‐FDCT‐20241114001). All animal experiments were conducted in accordance with the 3Rs principles (Replacement, Reduction, Refinement) and the ARRIVE guidelines 2.0.

### 5.3 Animal Models

To study cholestatic liver diseases, we generated the BDL mice (C57BL/6J mice 6‐ to 8‐week‐old, male) and purchased *Mdr2* ^−/−^ mice (7‐week‐old, male) from Cyagen (Guangzhou, China), and simultaneously treated the mice with metformin (Met, 100 mg/kg, daily) by intraperitoneal injection. *Hk1* ^fl/fl^ mice were generated by inserting Loxp sequences on both sides of exon 2 of the *Hk1* gene. The method for specific knockout of *Hk1* in cholangiocytes involved crossing 8‐week‐old *Hk1* ^fl/fl^ female mice with *Hk1* ^fl/+^; Krt19‐CreERT2 male mice. According to Mendelian inheritance laws, approximately one‐fourth of the embryos achieved specific knockout of *Hk1* in cholangiocytes. Similarly, to further confirm whether HK1 is required for the therapeutic effects of metformin, Krt19‐CreERT2*Hk1* ^fl/fl^ mice (C57BL/6J mice 8‐week‐old, male) were administered metformin (Met, MCE, HY‐17471A, 100 mg/kg/day) for 7 days following BDL. Mice were randomly assigned to experimental groups. sham+PBS: *n* = 6, sham+Met: *n* = 6, BDL+PBS: *n* = 13, BDL+Met: *n* = 15. WT+PBS, *n* = 10, *Mdr2* ^−/−^+PBS: *n* = 10, *Mdr2* ^−/−^+Met: *n* = 13. *Hk1* ^fl/fl^ (sham+PBS: *n* = 6, BDL+PBS: *n* = 15, BDL+Met: *n* = 17) and Krt19CreERT2*Hk1* ^fl/fl^ (sham+PBS: *n* = 6, BDL+PBS: *n* = 15, BDL+Met: *n* = 15). No a priori inclusion or exclusion criteria were set for animals or data points. All animals that successfully underwent the experimental procedures were included in the final analysis. Following the treatment, the mice were weighed and sacrificed to collect blood and livers. Group allocation and all experimental procedures were performed by the same investigator.

### 5.4 Establishment of Cholangiocyte Organoids

Cell extraction was performed as previously described [60]. Liver tissue from mice and BA patients were digested into single cells on Single Cell Suspension Dissociator (RWD, DSC‐400), and EpCAM positive cells were sorted following the manufacturer's protocol (Miltenyi Biotec, 130‐061‐101, human/130‐105‐958, mouse). EpCAM positive cells were then mixed with Matrigel (Corning Biocoat, 356231) and seeded onto 24‐well plates to generate organoids. P0 cholangiocyte organoids were collected for Bulk RNA sequencing. See Supporting Information Materials and Methods for details on the cholangiocyte organoids generation, passaging, freezing, and composition of medium.

### 5.5 Mouse Cholangiocyte Organoids Treatment

For inhibitor experiments, mouse Cho‐Orgs were seeded at a density of 1 × 10^4^ cells per well in 24‐well plates and cultured for 24 h to allow stable growth. Organoids were then treated with metformin (100 µM) alone or in combination with the ACLY inhibitor NDI‐091143 (1, 5, 10, 20 µM; MCE, HY‐127111) or the MOF inhibitor MG149 (1, 20, 50, 100 µM; MCE, HY‐15887) for 7 days. MG149 is a MYST family histone acetyltransferase inhibitor that targets MOF (KAT8/MYST1), the primary enzyme responsible for H4K16ac deposition, as well as Tip60 (KAT5), with modest potency (IC_50_ = 47 µM and 74 µM, respectively). Control groups received an equivalent volume of DMSO. The culture medium was replaced every 3 days with freshly prepared drugs. After treatment, organoids were harvested for staining and morphological assessment. All experiments were performed in triplicate with independent organoid cultures.

### 5.6 Treatment of Human Cholangiocyte Organoids With metformin

Metformin (MCE, HY‐17471A) was added to suspension of organoids derived from BA (final concentrations: 100 µM in PBS). The medium was changed with corresponding concentrations of metformin (100 µM) and control (PBS) every 3 days.

### 5.7 Immunofluorescence (IF) Staining

For liver tissues and organoids, after deparaffinized and hydrated, the 4.5‐µm sections were incubated with primary antibodies against CK19 (dilution, 1:250), CK7 (dilution, 1:100), ALB (dilution, 1:100), AFP (dilution, 1:100), vimentin (dilution, 1:100), COL1A1 (dilution, 1:100), αSMA (dilution, 1:200), LGR5 (dilution, 1:100), HK1 (dilution, 1:100), EpCAM (dilution, 1:1000), KI67 (dilution, 1:1000) and FN1 (dilution, 1:100) at 4°C overnight. Later, cells and sections were incubated with Alexa Fluor 594 Goat anti‐rabbit secondary antibody (dilution, 1:500) and Alexa Fluor 488 Goat anti‐mouse secondary antibody (dilution, 1:500) at least 1 h. DAPI was counterstained to mark nucleus. Finally, images were captured using MICA confocal laser scanning microscope (Leica, Germany). Detailed information regarding the antibodies is provided in the Supporting Information.

### 5.8 Statistical Analysis

All data are presented as mean ± SD as indicated. For human BA organoid experiments (Figure 6), each biological replicate represents organoids derived from an independent donor (*n* = 3). For each donor, technical triplicates were averaged prior to statistical analysis, and the donor‐derived averages were used as the biological unit (*n* = 3). For inhibitor experiments, *n* = 3 independent biological replicates (independent organoid cultures) were used; technical triplicates were averaged per biological replicate prior to analysis. Normality was assessed by the Shapiro–Wilk test where feasible; however, we acknowledge that with small sample sizes (*n* = 3), the power to detect deviations from normality is limited. Accordingly, we also performed non‐parametric tests (Mann–Whitney *U* test for two‐group comparisons, and Kruskal–Wallis with Dunn's post hoc correction for multiple‐group comparisons), which do not assume normality and yielded significance patterns consistent with parametric tests. For comparisons between two groups, two‐tailed Student's *t*‐test was applied. For multiple‐group comparisons, one‐way ANOVA followed by Dunnett's post hoc test was used for comparisons to the control group. Log‐rank (Mantel–Cox) test was utilized for survival analysis. ns, not significant. *p < 0.05; **p < 0.01; ***p < 0.001; ****p < 0.0001.

## Author Contributions

Conceptualization, methodology, validation, formal analysis, data curation, writing – original draft: Yingyu Liang. Methodology: Yongqin Ye, Haoran Lian, Zhejun Song, Xiaowen Bao, Yue Peng, Wenxia Zhang, Qingyu Lu, Weilu Wang, and Yiling Chen. Methodology and funding acquisition: Vincent Chi Hang Lui. Methodology, writing – review and editing: Fotios Sampaziotis. Conceptualization, data curation, funding acquisition, supervision, writing – review and editing: Yan Chen and Paul Kwong Hang Tam. All authors have read and approved the final manuscript.

## Funding

This work was supported by Macau Science and Technology Development Fund (FDCT No. 0086/2022/A, 0097/2022/A2, 0011/2023/AKP, and 0010/2025/AIJ) and Theme‐based Research Scheme (T12‐712/21‐R) from the Hong Kong.

## Ethics Statement

The clinical studies were approved by the Research Ethics Committee of the Third Affiliated Hospital of Sun Yat‐sen University (Approval Number: RG2024‐149‐01), with the need for patient approval and/or informed consent waived due to the retrospective nature of the study. All animal experiments were performed after institutional review by the Animal Ethics Committee of the Macau University of Science and Technology (MUST‐FDCT‐20241114001).

## Conflicts of Interest

The authors declare no conflicts of interest.

## Supporting information




**Supporting File**: mco271143‐sup‐0001‐SuppMat.pdf

## Data Availability

The data that support the findings of this study are available from the corresponding author upon reasonable request. The data that support the findings of this study are openly available in CNCB‐NGDC at https://ngdc.cncb.ac.cn/gsa‐human/, reference number HRA014387, HRA014383, and at https://ngdc.cncb.ac.cn/gsa/, reference number CRA031796 and CRA031780.
