# Supplementary material for: Metformin Promotes Cholangiocyte Regeneration in Cholestatic Fibrosis via HK1‐Glycolysis‐Dependent Epigenetic Activation of LGR5
Source: MedComm (2020). 2026 Nov 13;7(11):e71143. doi: 10.1002/mco2.71143 (PMC13761298; doi:10.1002/mco2.71143)
Supplement: Supplementary file 1 [file MCO2-7-e71143-s001.pdf]

Metformin promotes cholangiocyte regeneration in cholestatic fibrosis via HK1-glycolysis-dependent epigenetic activation of LGR5

Running title: Metformin/HK1-glycolysis activates LGR5

Yingyu Liang[1], Yongqin Ye[2], Haoran Lian[1], Zhejun Song[1], Xiaowen Bao[2], Yue Peng[2], Wenxia Zhang[2], Qingyu Lu[2], Weilu Wang[1], Yiling Chen[1], Vincent Chi Hang Lui[3], Fotios Sampaziotis[4], Paul Kwong Hang Tam[1,2,5,*], Yan Chen[2,5,*]

[1]School of Pharmacy, Faculty of Medicine, Macau University of Science and Technology, Macau 999078, China

[2]Faculty of Medicine, Macau University of Science and Technology, Macau 999078, China

[3]Department of Surgery, Li Ka Shing Faculty of Medicine, The University of Hong Kong, Hong Kong 999077, China

[4]Wellcome–MRC Cambridge Stem Cell Institute, Cambridge, United Kingdom

[5]Precision Regenerative Medicine Research Centre, Medical Sciences Division, Macau University of Science and Technology, Macau 999078, China

*Correspondence author: Yan Chen, Room: 209f, Praia Park Block R Coloane, Macau, China, Tel: +853 88973318, chenyan@must.edu.mo. and Paul Kwong Hang Tam, Room: N511, Block N, Avenida Wai Long, Taipa, Macau, China, Tel: +853 88972833 pkhtam@must.edu.mo

Table of contents

Supplementary Materials and Methods..3

Supplementary Figures..8

Supplementary Tables..24

Supplementary References..25

Supplementary Materials and Methods

Establishment of Cholangiocyte Organoids

Liver-tissue derived cholangiocyte organoids were generated following previously published protocol[1,2]. The cell supernatant was filtered through a 40 μm cell strainer and quantified. The filtered suspension was centrifuged at 580g (4℃) for 10 minutes. After supernatant removal, the cell pellet was resuspended in 100 μL of magnetic bead incubation buffer. CD326 (EpCAM) magnetic beads were added at a ratio of 10 μL per 10^7 cells, followed by 15-minute incubation at 4℃ protected from light. The reaction was terminated by adding 900 μL of magnetic bead incubation buffer. The magnetic separation system was assembled with pre-washed LS columns and collection tubes for both EpCAM-negative and -positive fractions. Columns were equilibrated with 3 mL of column buffer, and the cell-bead mixture was applied as the buffer meniscus reached the column surface. Following sample application, three sequential 3 mL washes with column buffer were performed. The flow-through containing the EpCAM-negative fraction was collected for subsequent hepatic stellate cells isolation. For EpCAM-positive cell recovery, the column was removed from the magnetic separator and placed onto a fresh collection tube. Positive cells were eluted by flushing the column with a 5 mL column buffer using the plunger supplied with the column. The eluted cell suspension was centrifuged at 300g (4℃) for 10 minutes. The pellet was resuspended in 1 mL ice-cold advanced DMEM/F12 basal medium for cell counting. After another centrifugation cycle (300g, 4℃, 10 minutes), the cell pellet was resuspended in Matrigel™ at a 1:1000 matrix-to-cell ratio. The cell-Matrigel™ suspension was plated and allowed to polymerize before adding 500 μl of cholangiocyte organoid medium per well. Organoid medium was based on Advanced DMEM/F12 (12634028, Gibco, USA) supplemented with Penicillin/ Streptomycin/ Fungizone (C100C8, NCM Biotech, China), GlutaMax (35050-061, Gibco, USA), 25 mM HEPES (15630-080, Gibco, USA), 1% N2 (17502001, Gibco, USA), 1% B27 (17504001, Gibco, USA), 1.25 mM N-Acetylcysteine (A9165, Sigma, Germany), 10 nM gastrin (G9145, Sigma, Germany),

50 ng/ml EGF (PMG8043, Gibco, USA), 100 ng/ml FGF10 (100-26, PeproTech, USA), 25 ng/ml HGF (100-39, PeproTech, USA), 10 mM Nicotinamide (N0636, Sigma, Germany), 5 μM A83-01 (72022, stemcell, Canada), 10 μM Forskolin (B1421, Apexbio, USA), R-Spondin 1/RSPO1 Protein (TMPY-03197, Topscience Co. Ltd. ,China), 100 ng/ml Noggin (250-38, Peprotech, USA), 100 ng/ml Wnt3a (31520, Peprotech, USA), 10 μM ROCK inhibitor Y-27632 (S1049, Selleckchem, USA). Medium was changed once every three days.

Passaging and freezing of organoids

Cultures were passaged when organoids grew beyond the size limit following previously published protocol[1,2]. For passaging, the organoids along with the Matrigel™ were scraped from the bottom and pipetted up and down to break up organoids into small pieces in 1 ml Advanced DMEM/F12 medium. The disrupted organoids were next transferred to 15 ml tubes containing 5-10 ml of Advanced DMEM/F12 medium. The samples were centrifuged at 4℃ at 300 g for 10 min after keeping in ice for 30 min to dissolve the Matrigel™. The supernatant was aspirated, and the cell pellets were resuspended in 50 μl of Matrigel™. About 500 μl of prewarmed medium was then added per well after solidification of Matrigel™. The medium was changed every alternate day for 1 to 2 weeks. For freezing of organoids, instead of mixing with Matrigel™ in the above step, 1 ml of CELLSAVING (C40100, NCM Biotech, China) was added followed by transfer to a 2 ml freezing tube. The tube was kept at -80℃ for 2-3 days and transferred to liquid nitrogen for slow freezing to avoid cell damage. Each piece of frozen organoids could be grown back to a complete organoid structure within 1-2 days. For resumed culturing of frozen organoids, the incubated frozen organoids were quickly thawed at 37℃ in a water bath. The content of the tube was added drop by drop to a tube containing 5 ml of Advanced DMEM/F12 medium and centrifuged at 300 g for 10 min. The Matrigel™ was then resuspended and 500 μl of organoid medium per well was added after solidification of the Matrigel™. Medium was changed once every three days.

CUT & Tag

The CUT & Tag assay was conducted following the protocol provided in the CUT & Tag Assay Kit manual (TD904-01, Vazyme, China). Briefly, after conA bead activation, 95 μL of wash buffer was added to the samples and incubated for 10 minutes at room temperature. The H4K16ac antibody-bead complexes were then incubated on a rotator for 2 hours at room temperature. Following secondary antibody incubation, the samples were mixed with a 1:120 dilution of pA/G-Tnp5 transposase (0.033 mM) and incubated for 1 hour. Subsequently, the samples were subjected to tagmentation in 40 μL of tagmentation buffer at 37°C for 1 hour. DNA extraction was performed according to the manufacturer's instructions, and the purified DNA was dissolved in 20 μL of nuclease-free water. For library amplification, P5/P7 adapters (TD202, Vazyme, China) and 2×CAM were added, followed by PCR. PCR cleanup was carried out by adding 2 volumes of DNA clean beads, and the final libraries were eluted in 30 μL of nuclease-free water. The libraries were pooled and sequenced using 150 bp paired-end reads on the NovaSeq platform.

Liver histological and immunohistological staining

After fixed with 4% formalin for one week, liver specimens embedded in paraffin and cut into 4.5-μm sections for subsequent experiments. To examine hepatic morphology and evaluate liver fibrosis, hematoxylin and eosin (H&E), Masson's trichrome staining, and Sirus red staining were performed, respectively. The sections were deparaffinized in xylene three times, hydrated by ethyl alcohol and respectively stained using H&E Staining kit and Masson Stain Kit. For Sirus red staining, the sections were hydrated and stained with Sirus red staining solution for 10 minutes, following the protocol of the Sirus red staining kit. For immunohistochemistry (IHC) staining, the sections were incubated with 3% H_2O_2 for 10 minutes to quench the endogenous peroxidase activity. Following blocking with 5% BSA reagent, the slides were incubated with primary antibodies against H4K16ac (dilution, 1:100) and KI67 (dilution, 1:300) at 4 ℃ overnight. After washed and incubated with goat anti-mouse/rabbit IgG HRP polymer secondary

antibody, the slides were imaged by Mica (Leica, Germany). CK19 (Zenbio, R24060, dilution, 1:100), CK7 (Zenbio, 250022, dilution, 1:100) , ALB (Proteintech, 16475-1-AP, dilution, 1:100), AFP (Proteintech, 14550-1-AP, dilution, 1:100), Vimentin (Zenbio, R22775, dilution, 1:100), COL1A1 (Zenbio, 501352, dilution, 1:100), αSMA (CST, 48938S, dilution, 1:200), LGR5 (Zenbio, R380973, dilution, 1:100), HK1 (Proteintech, 68419-1-Ig, dilution, 1:100), KI67 (Zenbio, R381101, dilution, 1:100), PCNA (Zenbio, R25294, dilution, 1:100), H4K16ac (Zenbio, 350088, dilution, 1:100), EpCAM (Zenbio, R24218, dilution, 1:1000), KI67 (Zenbio, R381101, dilution, 1:1000) and FN1 (Proteintech, 66042-1-Ig, dilution, 1:100).

Serum biochemical measurements

After resting at room temperature, serum was centrifuged at 3000 rpm for 15 minutes before usage. The levels of alanine aminotransferase (ALT), aspartate aminotransferase (AST), alkaline phosphatase (ALP), total bile acid(TBA), total bilirubin (TBIL), direct bilirubin (DBIL), albumin (ALB), and γ-glutamyl transferase (γ-GT) in serum were determined according to the manufacturer's instructions. The above kits were purchased from Nanjing Jiancheng Bioengineering Institute (Nanjing, China).

RNA isolation and real-time qPCR

In accordance with the manufacturer's protocol, the total RNA was isolated from organoids using TRIzol Reagent and measured using NanoDrop One Microvolume UV-Vis Spectrophotometer (Thermo, Waltham, USA). The cDNA was synthesized using PrimeScript™ RT reagent Kit with gDNA Eraser (RR047A, Takara, Japan) as per the manufacturer's instructions. The mRNA levels of targeted genes were quantified using qPCR. The list of primers in this research could be found in Table S1.

Detection of acetyl-CoA Secretion

Acetyl-CoA secretion levels in organoid culture supernatants were quantified using a commercially available Acetyl-CoA ELISA Quantitation Kit (D751001 , Sangon Biotech, China) according to the manufacturer's protocol. Briefly, culture supernatants were

collected after 24 hours of incubation and centrifuged at 300g for 10 minutes at 4℃ to remove cellular debris. Standards and samples were assayed in duplicate using 100 μL aliquots. The optical density was measured at 450 nm using a microplate reader, with wavelength correction set at 630 nm. Acetyl-CoA concentrations were determined by interpolation from the standard curve generated using recombinant mouse albumin standards. Three independent biological replicates were performed for each experimental condition.

RNA-sequencing analysis

Liver tissue RNA was extracted using TRIzol Reagent and quantified with a NanoDrop One Microvolume UV-Vis Spectrophotometer (Thermo, Waltham, USA). After cDNA synthesis and mRNA purification, cDNA fragments between 250-300 bp were selected for sequencing library production on an Illumina Novaseq platform. Quality control analyses were performed on the samples and normalization was done using edgeR software. The differentially expressed genes (DEGs) were analyzed for Gene set-enrichment (GSEA) analysis using the cluster Profiler R package.

Supplementary Figures

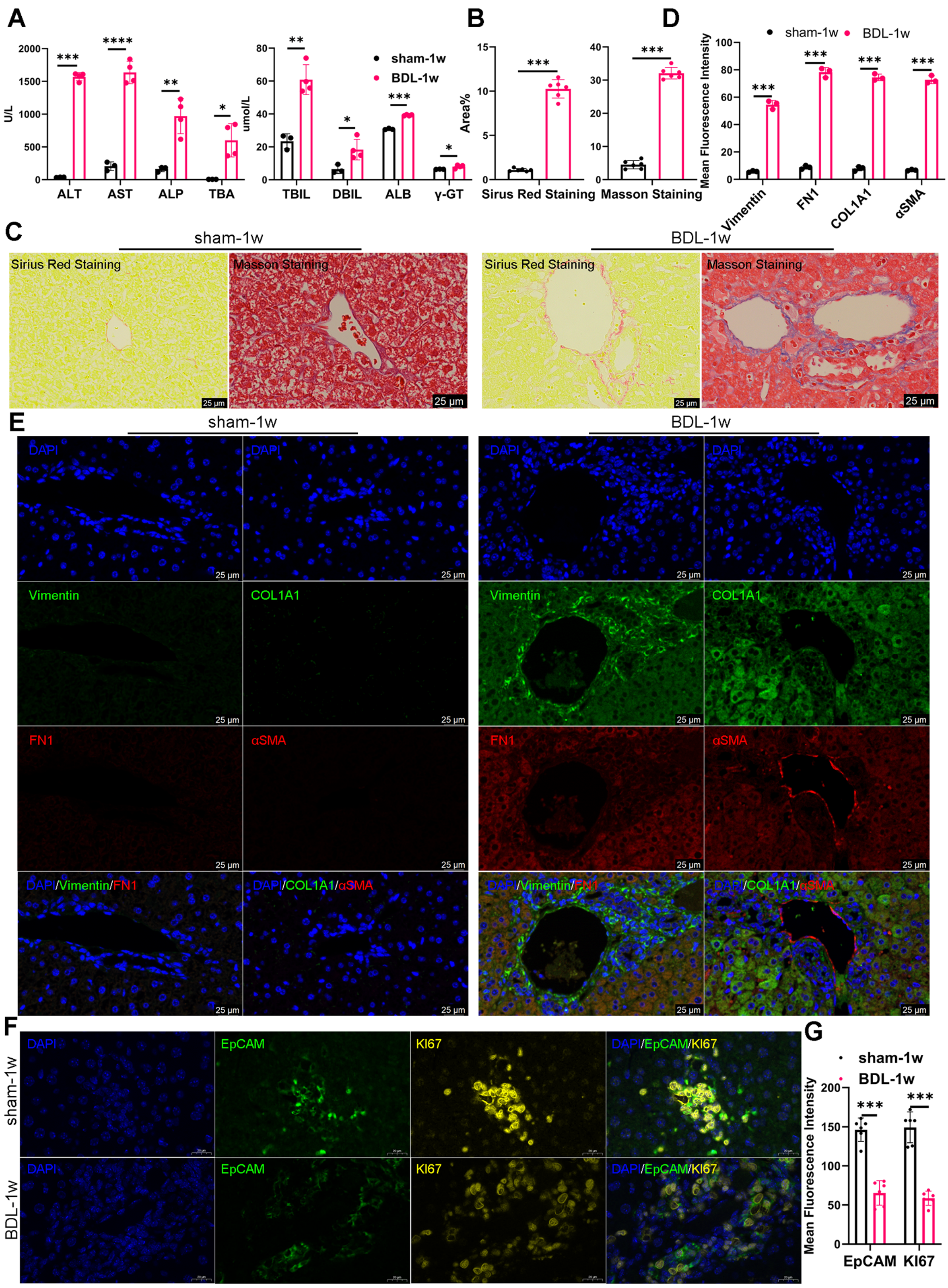

Figure S1. Elevated fibrotic level was observed in BDL mice. (A) The serum levels of ALT, AST, ALB, TBIL, DBIL, ALP, γ-GT and TBA. (B-C) Sirus red staining and Masson

staining of BDL-1w liver section. Scale bar=25μm. (D-E) The protein levels and quantification of Vimentin, FN1, a-SMA, and COL1A1 in BDL-1w liver tissues. Scale bar=25μm. (F-G) The protein levels and quantification of EpCAM and KI67 in BDL-1w liver tissues. Scale bar=200μm. *p<0.05, **p<0.01, ***p<0.001, ****p<0.0001.

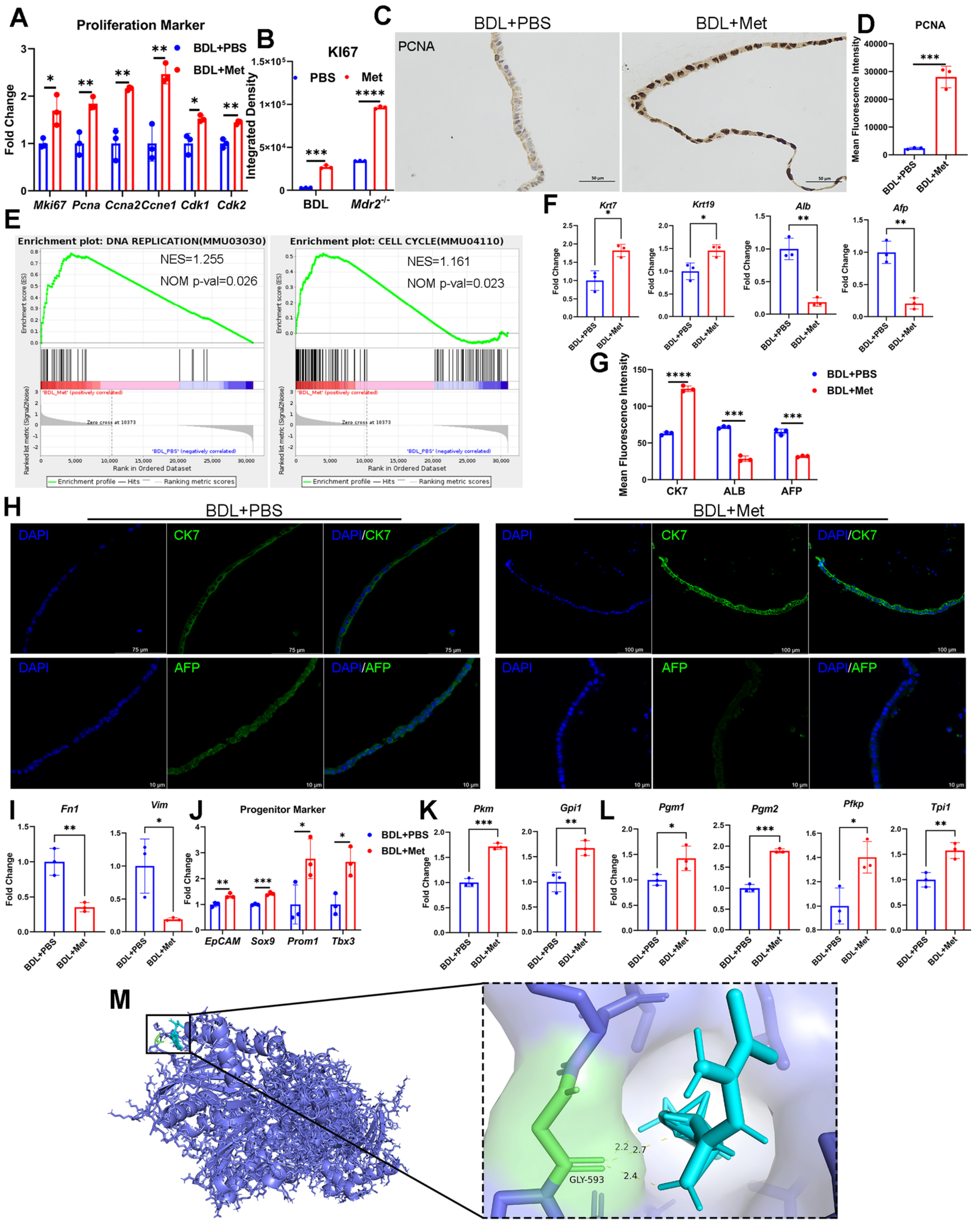

Figure S2. Metformin promotes regeneration and glycolysis on Cho-Orgs in BDL mice. (A) The mRNA level of proliferation markers in Cho-Orgs. (B) Quantification of KI67 in Cho-Orgs. (C-D) The protein levels and quantification of PCNA in Cho-Orgs section. Scale bar=50μm. (E) GSEA analysis of DNA REPLICATION and CELL CYCLE. (F) The mRNA level of *Krt7*, *Krt19*, *Alb* and *Afp* in Cho-Orgs. (G-H) The protein levels

and quantification of CK7 and AFP in Cho-Orgs section. Scale bar=10, 75, 100μm. (I) The mRNA level and quantification of staining on *Fn1*, *Vim* in Cho-Orgs. (J) The mRNA level of progenitor markers *EpCAM*, *Sox9*, *Prom1*, *Tbx3* in Cho-Orgs. (K-L) The mRNA level of *Pfkp*, *Pgm1*, *Pgm2*, *Pkm* and *Tpi1* in Cho-Orgs. (M) Autodocking analysis between metformin and HK1. **p*<0.05, ***p*<0.01, ****p*<0.001, *****p*<0.0001.

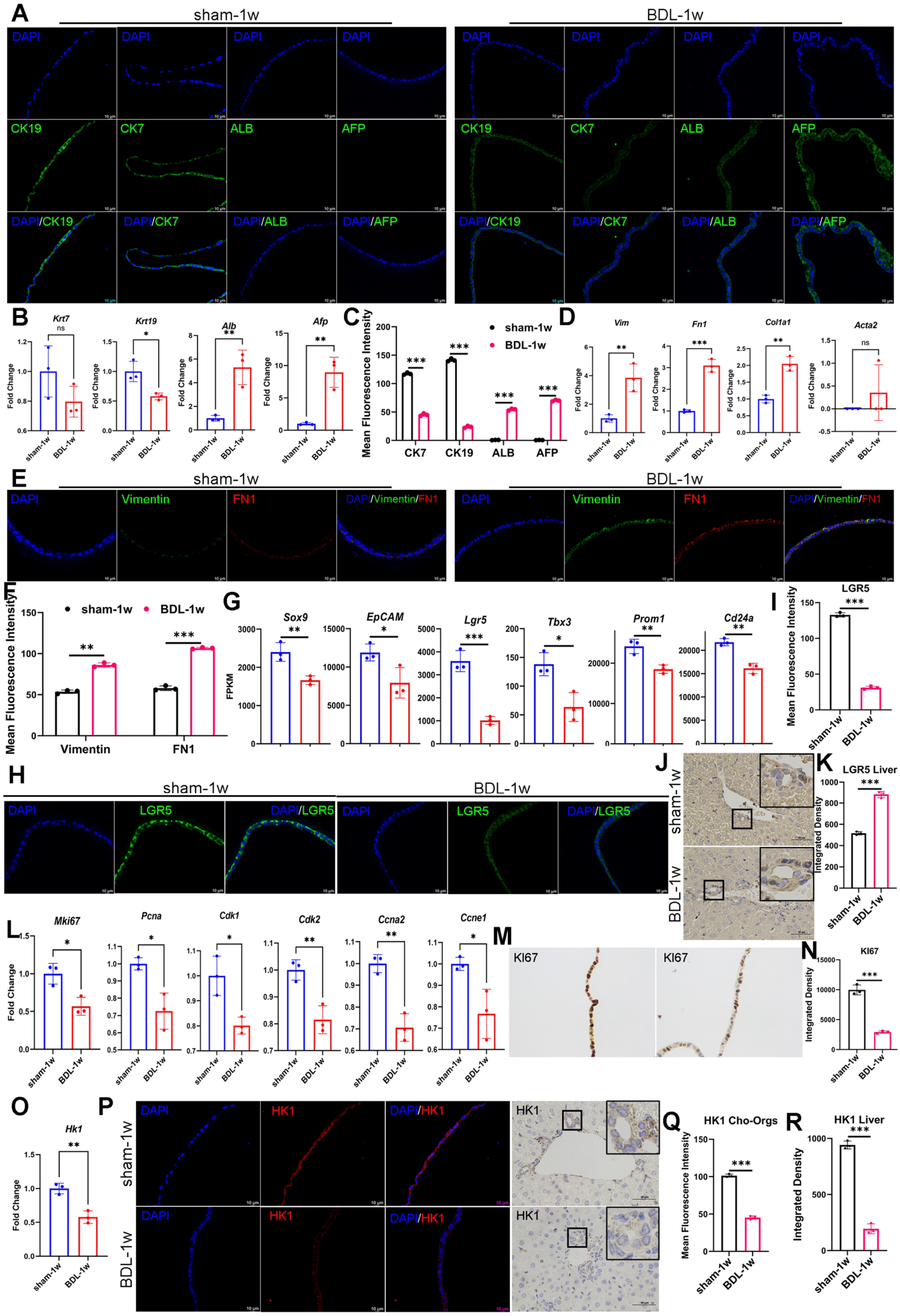

Figure S3. Elevated EMT level and impaired cholangiocyte stemness were observed on Cho-Orgs in BDL mice. (A-C) The levels of CK7, CK19, ALB and AFP

in Cho-Orgs section. Scale bar=10, 50μm. (D) The mRNA level of *Fn1*, *Vim*, *Col1a1* and *Acta2* in Cho-Orgs. (E-F) The protein levels of Vimentin, FN1 in Cho-Orgs section. Scale bar=10μm. (G) The mRNA level of *Lgr5*, *Sox9, EpCAM, Tbx3, Prom1, Cd24a* in Cho-Orgs. (H-K) The protein levels of LGR5 in Cho-Orgs and liver section. Scale bar=10, 50μm. (L) The mRNA level of *Mki67*, *Pcna*, *Cdk1*, *Cdk2*, *Ccna2* and *Ccne1* in Cho-Orgs. (M-N) The protein levels of KI67 in Cho-Orgs section. Scale bar=50μm. (O-R) The levels of HK1 in Cho-Orgs and liver section. Scale bar=10, 50μm. **p*<0.05, ***p*<0.01, ****p*<0.001, *****p*<0.0001.

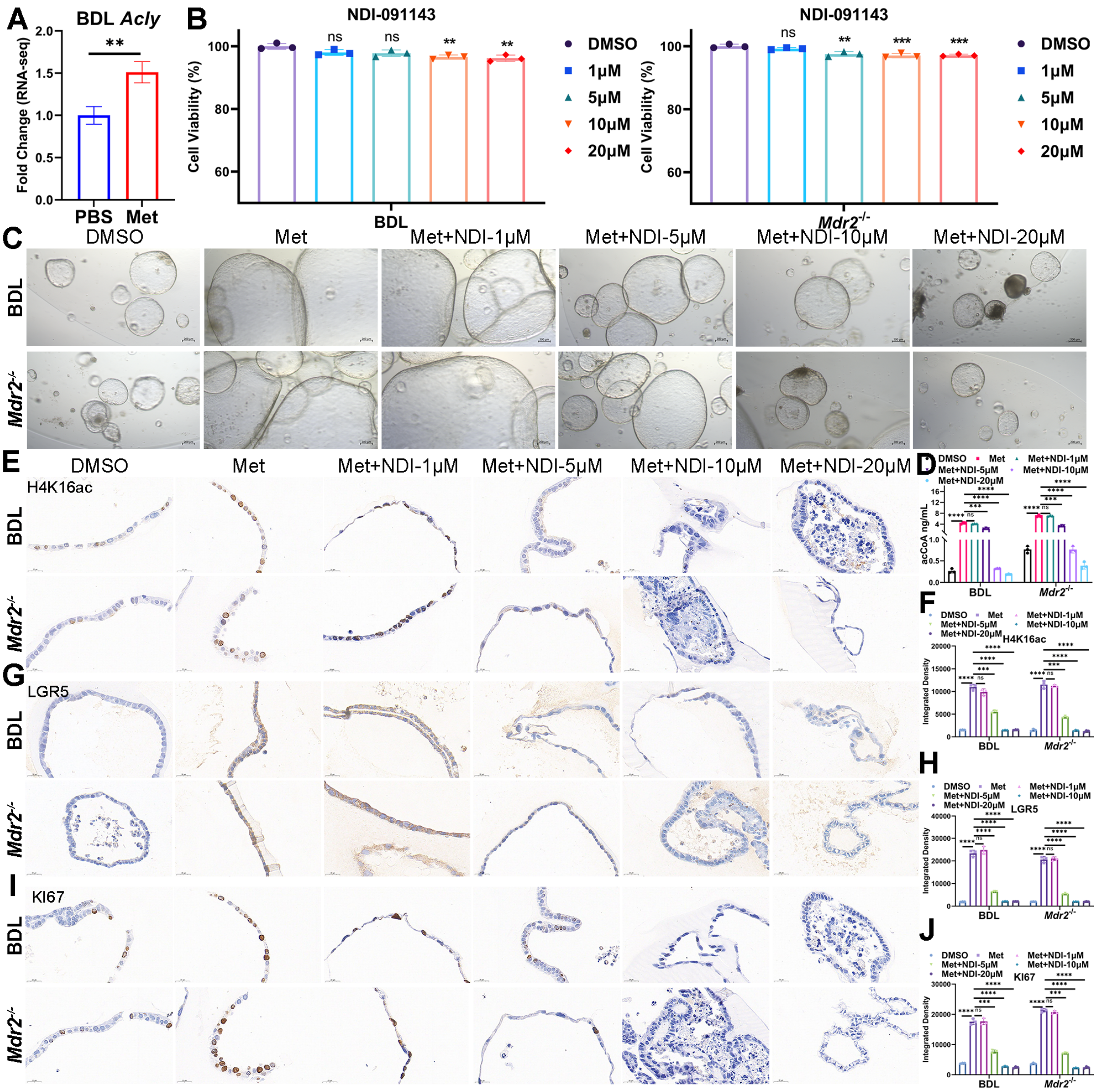

Figure S4. The effects of ACLY inhibitor NDI-091143 on BDL-derived Cho-Orgs. (A) mRNA level of *Acly* on Met-treated BDL Cho-Orgs. (B) CCK8 assays of NDI-091143

(1, 5, 10, 20 μM) for 7 days on Cho-Orgs. (C) Representative images of Met- or in combination with NDI-091143-treated Cho-Orgs. (D) The level of acCoA of supernatant from Met- or in combination with NDI-091143-treated Cho-Orgs. (E-F) Staining and quantification of H4K16ac of Met- or in combination with NDI-091143-treated Cho-Orgs. Scale bar=20μm. (G-H) Staining and quantification of LGR5 of Met- or in combination with NDI-091143-treated Cho-Orgs. Scale bar=20μm. (I-J) Staining and quantification of KI67 of Met- or in combination with NDI-091143-treated Cho-Orgs. Scale bar=20μm. n = 3 independent biological replicates (independent organoid cultures). Technical triplicates were averaged per biological replicate prior to analysis. *p<0.05, **p<0.01, ***p<0.001, ****p<0.0001.

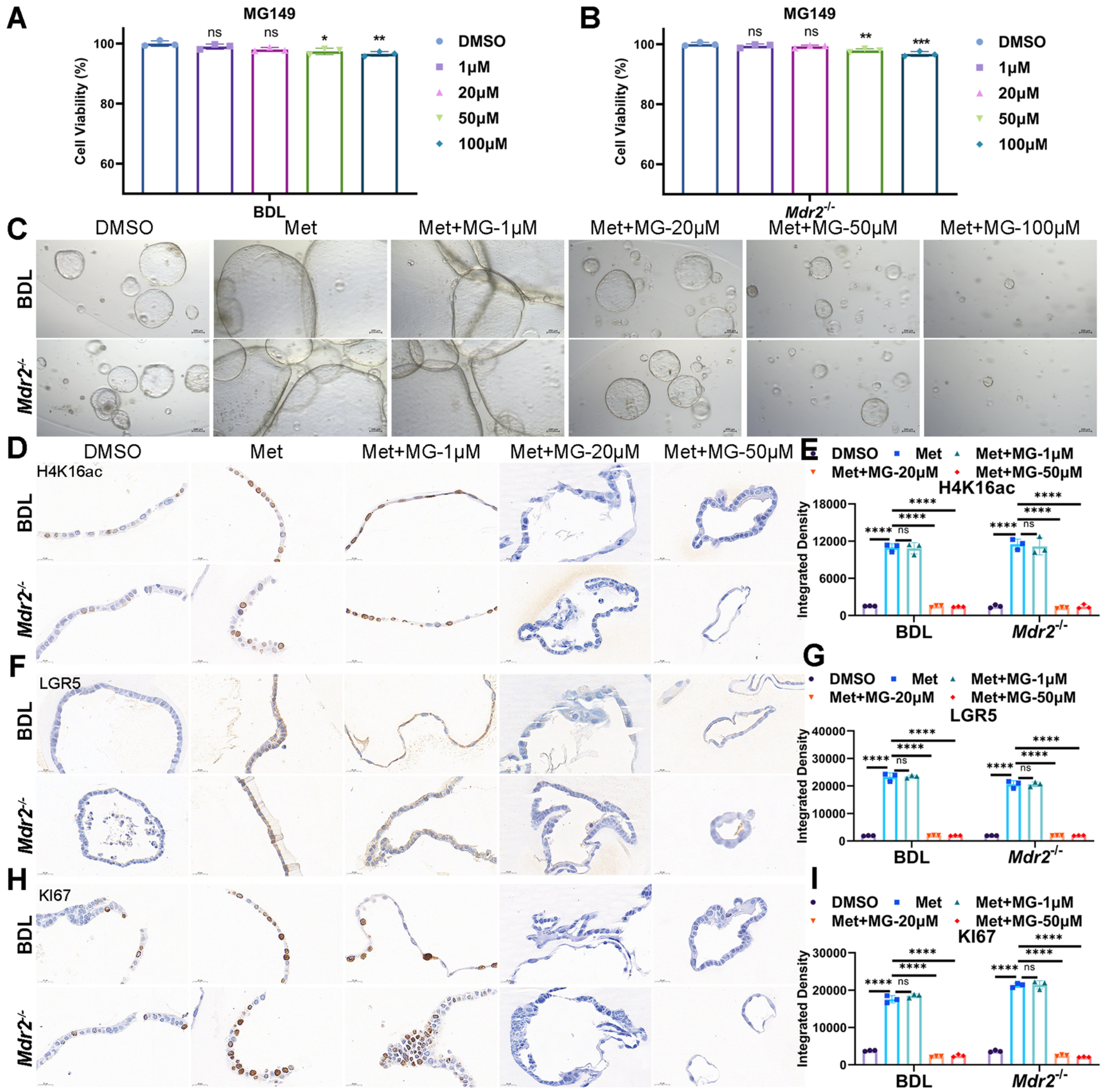

Figure S5. The effects of H4K16ac inhibitor MG149 on BDL-derived Cho-Orgs. (A-B) CCK8 assays of MG149 (1, 20, 50, 100 μM) for 7 days on Cho-Orgs. (C) Representative images of Met- or in combination with MG149-treated Cho-Orgs. (D-E) Staining and quantification of H4K16ac of Met- or in combination with MG149-treated Cho-Orgs. Scale bar=20μm. (F-G) Staining and quantification of LGR5 of Met- or in combination with MG149-treated Cho-Orgs. Scale bar=20μm. (H-I) Staining and quantification of KI67 of Met- or in combination with MG149-treated Cho-Orgs. Scale bar=20μm. n = 3 independent biological replicates (independent organoid cultures). Technical triplicates were averaged per biological replicate prior to analysis. *p<0.05, **p<0.01, ***p<0.001, ****p<0.0001.

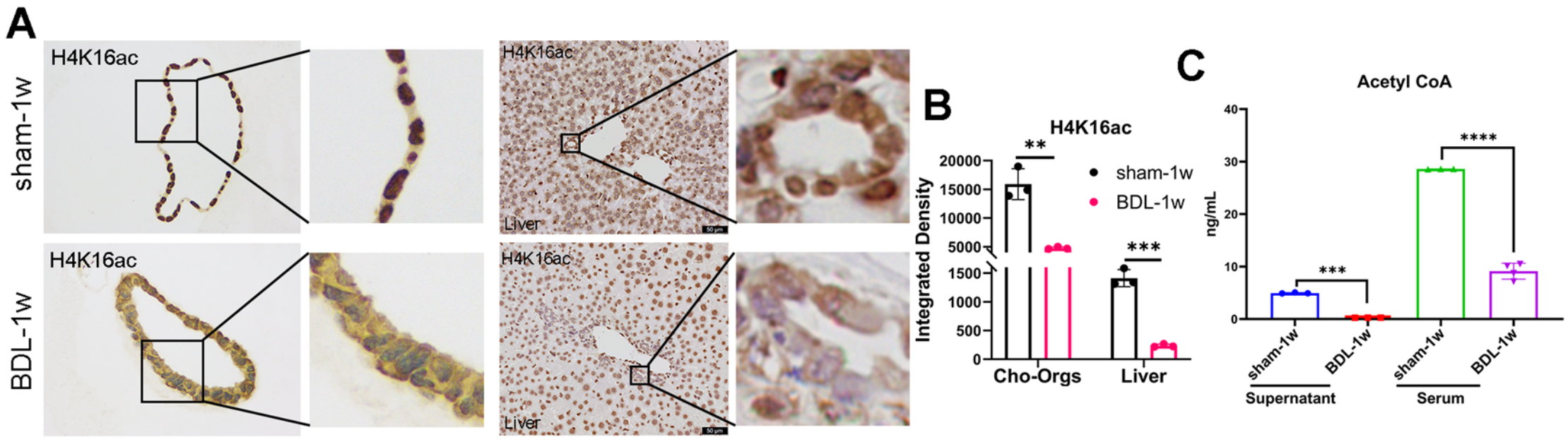

Figure S6. Decreased acetyl-CoA and H4K16ac level were observed on Cho-Orgs in BDL mice. (A-B) Staining and quantification of H4K16ac of Cho-Orgs and liver section. Scale bar=50μm. (C) ELISA detection of acetyl-CoA in serum and organoid supernatant. *p<0.05, **p<0.01, ***p<0.001, ****p<0.0001.

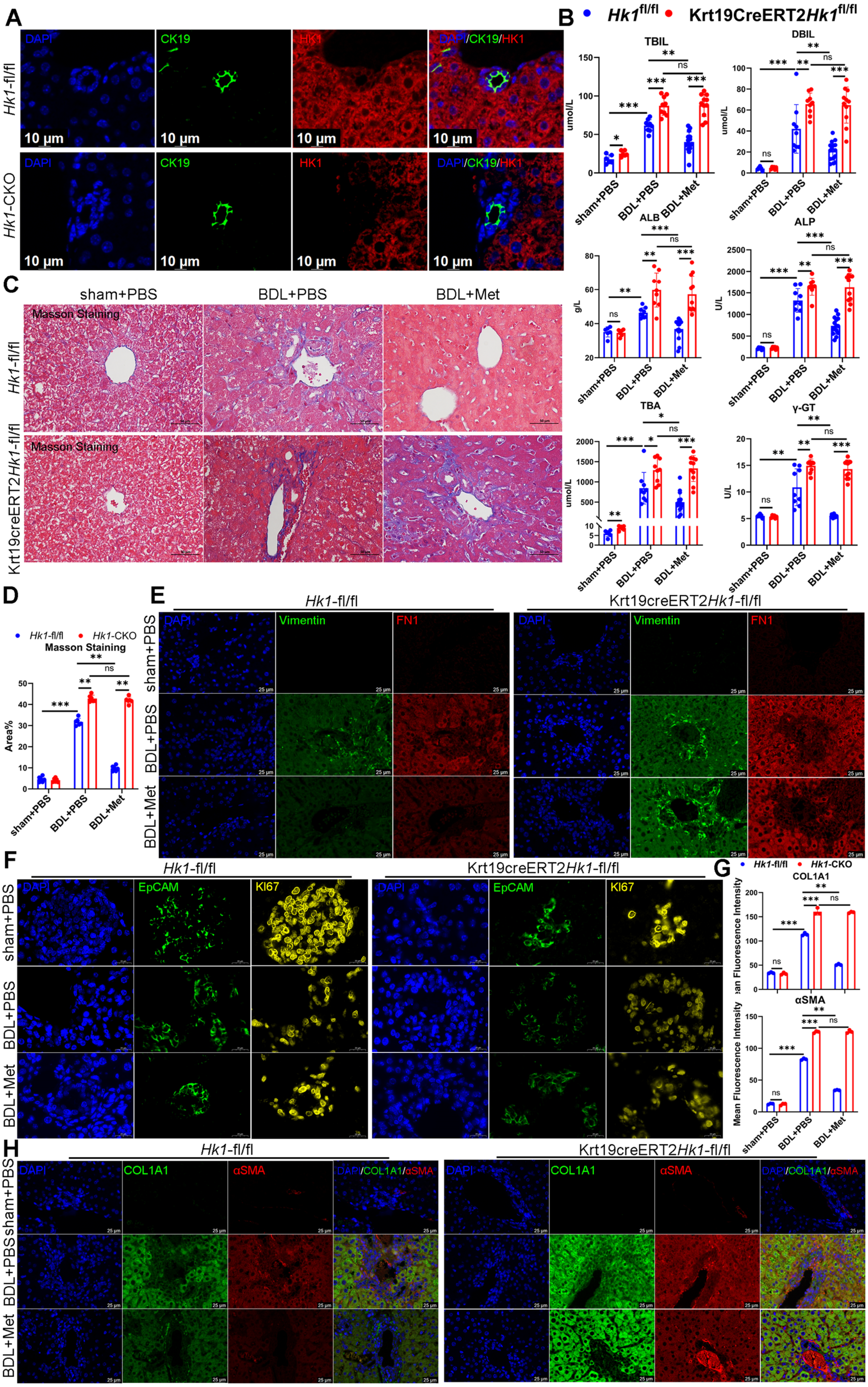
A
B
C
D
E
F
G
H
DAPI
CK19
HK1
DAPI/CK19/HK1
10 μm
Hk1-fl/fl
Hk1-CKO
Hk1fl/fl
Krt19CreERT2Hk1fl/fl
TBIL
DBIL
ALB
ALP
TBA
γ-GT
umol/L
g/L
U/L
sham+PBS
BDL+PBS
BDL+Met
Masson Staining
Krt19creERT2Hk1-fl/fl
Area%
Vimentin
FN1
EpCAM
KI67
COL1A1
αSMA
Mean Fluorescence Intensity
DAPI/COL1A1/αSMA
25 μm
ns
*
**
***

Figure S7. The anti-fibrotic effect of metformin is blocked by HK1 deficiency. (A) Staining to validate the efficiency of *Hk1* knockout. Scale bar=10μm. (B) TBIL, DBIL, ALB, ALP, TBA, GGT levels. (C-D) Masson staining of liver section. Scale bar=50μm. (E) Staining of FN1, Vimentin of liver tissues. Scale bar=25μm. (F) Staining of EpCAM, Ki67 of liver tissues. Scale bar=200μm. (G-H) The levels of COL1A1 and αSMA of liver tissues. Scale bar=25μm. **p*<0.05, ***p*<0.01, ****p*<0.001, *****p*<0.0001.

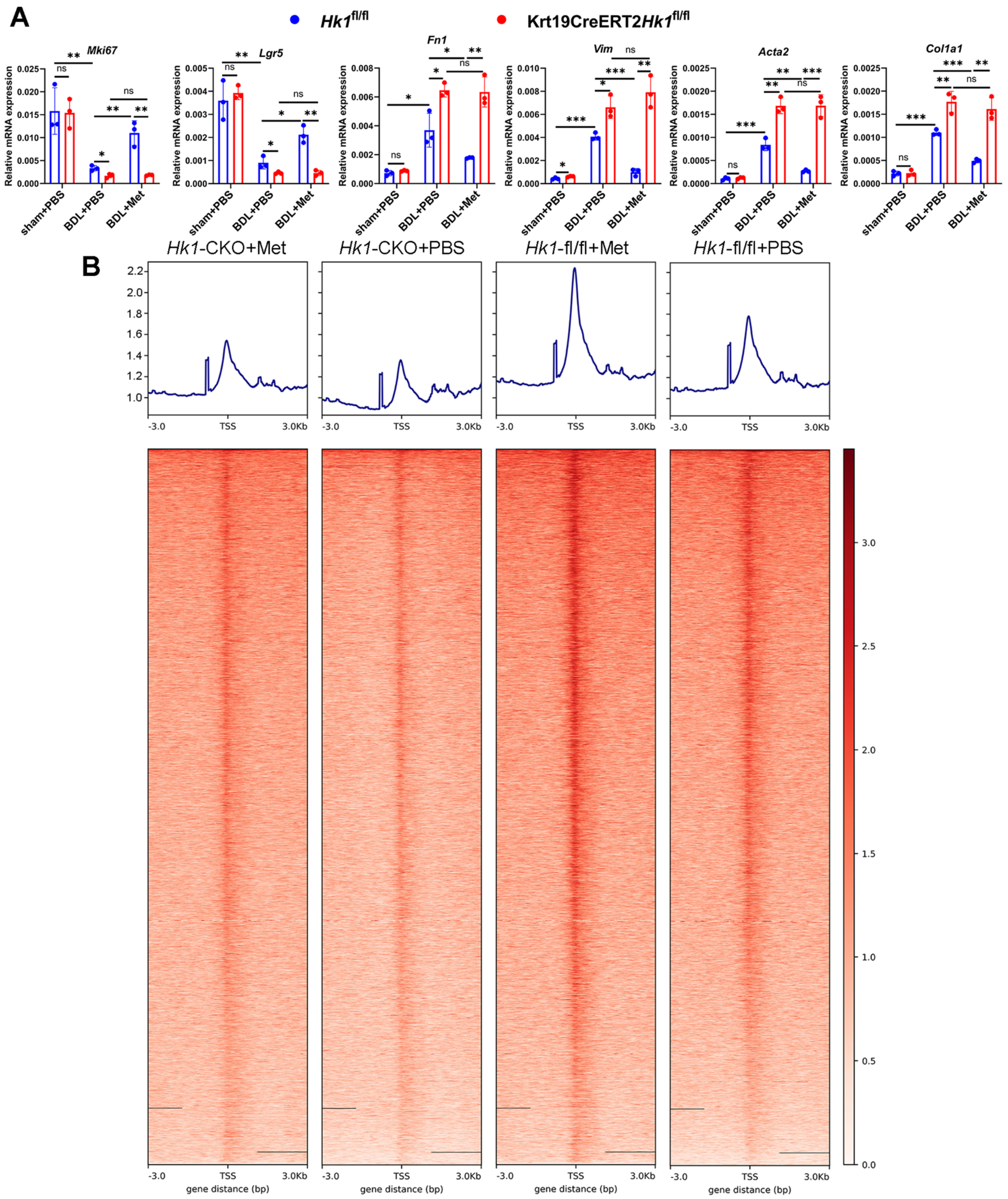

Figure S8. The proliferative, stemness-promoting and anti-fibrotic role of metformin are reversed by *Hk1* knockout. (A) mRNA level of *Mki67*, *Lgr5*, *Fn1*, *Vim*, *Acta2* and *Col1a1* in Cho-Orgs. (B) Heatmap of CUT & Tag on the binding between H4K16ac and gene TSS. *p<0.05, **p<0.01, ***p<0.001, ****p<0.0001.

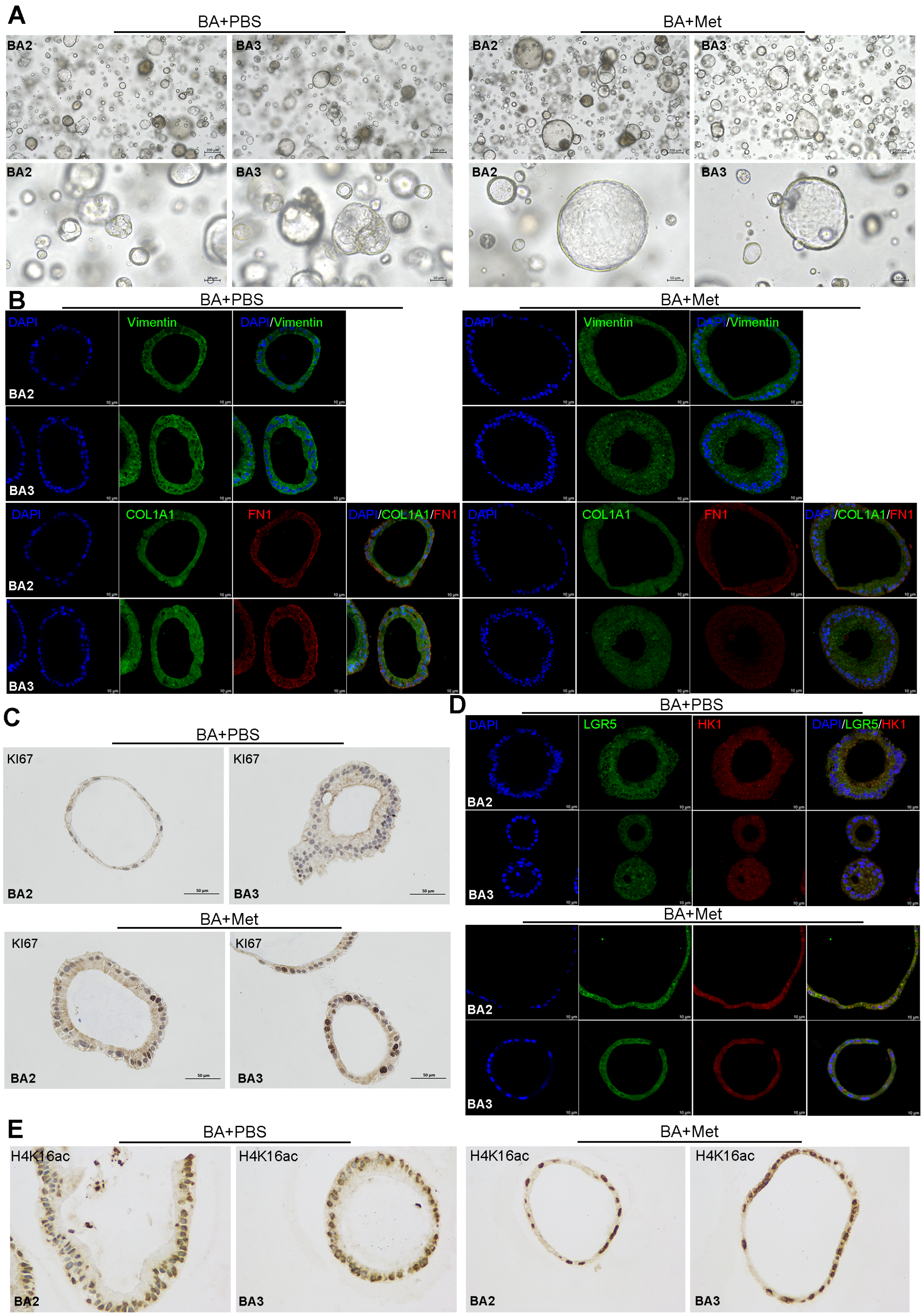

Figure S9. Metformin promotes stemness and reduces fibrosis of Cho-Orgs derived from human samples. (A) Representative images of metformin-treated BA

Cho-Orgs. Scale bar=50, 200μm. (B) Staining of COL1A1, FN1, Vimentin in metformin-treated BA Cho-Orgs. Scale bar=10μm. (C) Staining of KI67 in metformin-treated BA Cho-Orgs. Scale bar=50μm. (D) Staining of LGR5, HK1 in metformin-treated BA Cho-Orgs. Scale bar=10μm. (E) Staining of H4K16ac in metformin-treated BA Cho-Orgs. Scale bar=50μm. *p<0.05, **p<0.01, ***p<0.001, ****p<0.0001.

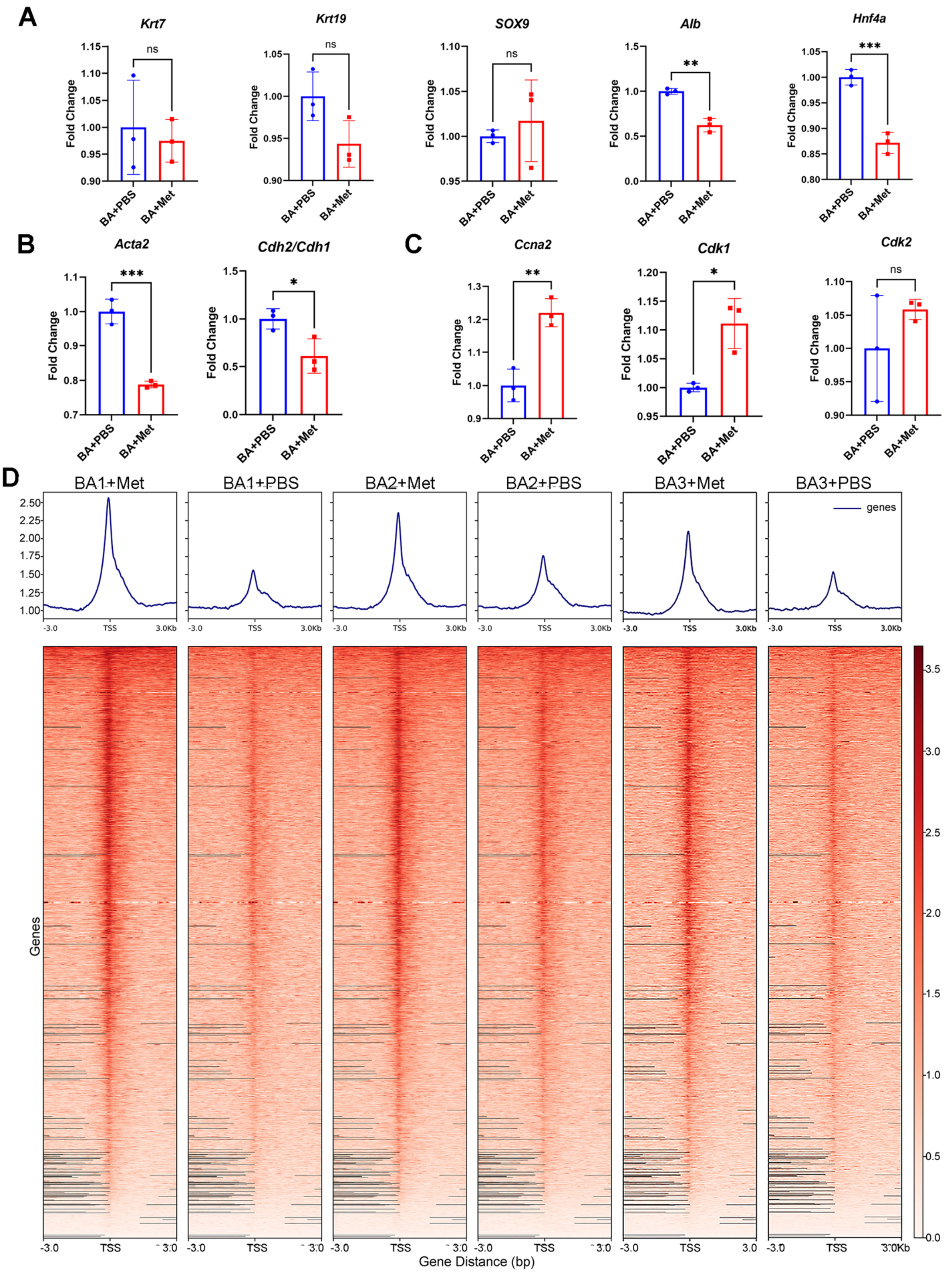

Figure S10. Metformin improves stemness and reduces fibrosis of Cho-Orgs derived from human samples. (A-C) mRNA level of *Krt7*, *Krt19*, *Sox9*, *Alb*, *Hnf4α*, *Acta2*, *Cdh2*, *Cdh1,* *Ccna2*, *Cdk1* and *Cdk2* in metformin-treated BA Cho-Orgs. (D) The heatmap of the binding between H4K16ac and genes of metformin-treated BA

Cho-Orgs. *$p<0.05$, **$p<0.01$, ***$p<0.001$, ****$p<0.0001$.

Supplementary tables

Table S1. Primer sequences used in qPCR.

Genes	Forward primer (5'-3')	Reverse primer (5'-3')
Acta2	GTCCCAGACATCAGGGAGTAA	TCGGATACTTCAGCGTCAGGA
Col1a1	GCTCCTCTTAGGGGCCACT	ATTGGGGACCCTTAGGCCAT
Actb	GGCTGTATTCCCCTCCATCG	CCAGTTGGTAACAATGCCATGT
Fn1	GCTCAGCAAATCGTGCAGC	CTAGGTAGGTCCGTTCCCACT
Mki67	ATCATTGACCGCTCCTTTAGGT	GCTCGCCTTGATGGTTCCT
Vim	CGGCTGCGAGAGAAATTGC	CCACTTTCCGTTCAAGGTCAAG
Lgr5	CCTACTCGAAGACTTACCCAGT	GCATTGGGGTGAATGATAGCA
Lgr5-CUT&Tag	GCTGCTGCACACAGCTG	CTCCTTTTTTAAAGCGCTCCA
Myod1-CUT&Tag	TGGGCTGGAAGGAAGAGATG	CCTGGCTAATGTTCAGGTGTG

References

1. Babu RO, Lui VCH, Chen Y, et al. Beta-amyloid deposition around hepatic bile ducts is a novel pathobiological and diagnostic feature of biliary atresia. *J Hepatol*. 2020;73(6):1391-1403. doi:10.1016/j.jhep.2020.06.012

2. Liang Y, Ye Y, Xie H, Lui VCH, Chen Y, Tam PKH. A robust mouse liver organoid platform enables sustained multicellular maturation and fibrosis modeling from a single tissue sample. *Sci Rep*. 2026;doi:10.1038/s41598-026-42990-2
